# An eye for a tooth: *Thylacosmilus* was not a marsupial “saber-tooth predator”

**DOI:** 10.7717/peerj.9346

**Published:** 2020-06-26

**Authors:** Christine M. Janis, Borja Figueirido, Larisa DeSantis, Stephan Lautenschlager

**Affiliations:** 1 School of Earth Sciences, University of Bristol, Bristol, United Kingdom; 2 Department of Ecology and Evolutionary Biology, Brown University, Providence, RI, United States of America; 3 Departamento de Ecología y Geología, Universidad de Málaga, Málaga, Spain; 4 Department of Biological Sciences, Vanderbilt University, Nashville, TN, United States of America; 5 Department of Earth and Environmental Sciences, Vanderbilt University, Nashville, TN, United States of America; 6 School of Geography, Earth & Environmental Sciences, University of Birmingham, Birmingham, UK

**Keywords:** Palaeobiology, Vertebrate palaeontology, Fossil, Evolution, Computational modelling, Saber-tooth ecomorphology, Dental microwear texture analysis, Finite element analysis, Canonical correspondence analysis

## Abstract

**Background:**

Saber-toothed mammals, now all extinct, were cats or “cat-like” forms with enlarged, blade-like upper canines, proposed as specialists in taking large prey. During the last 66 Ma, the saber-tooth ecomorph has evolved convergently at least in five different mammalian lineages across both marsupials and placentals. Indeed, *Thylacosmilus atrox*, the so-called “marsupial saber-tooth,” is often considered as a classic example of convergence with placental saber-tooth cats such as *Smilodon fatalis*. However, despite its superficial similarity to saber-toothed placentals, *T. atrox* lacks many of the critical anatomical features related to their inferred predatory behavior—that of employing their enlarged canines in a killing head strike.

**Methods:**

Here we follow a multi-proxy approach using canonical correspondence analysis of discrete traits, biomechanical models of skull function using Finite Element Analysis, and 3D dental microwear texture analysis of upper and lower postcanine teeth, to investigate the degree of evolutionary convergence between *T. atrox* and placental saber-tooths, including *S. fatalis*.

**Results:**

Correspondence analysis shows that the craniodental features of *T. atrox* are divergent from those of placental saber-tooths. Biomechanical analyses indicate a superior ability of *T. atrox* to placental saber-tooths in pulling back with the canines, with the unique lateral ridge of the canines adding strength to this function. The dental microwear of *T. atrox* indicates a soft diet, resembling that of the meat-specializing cheetah, but its blunted gross dental wear is not indicative of shearing meat.

**Conclusions:**

Our results indicate that despite its impressive canines, the “marsupial saber-tooth” was not the ecological analogue of placental saber-tooths, and likely did not use its canines to dispatch its prey. This oft-cited example of convergence requires reconsideration, and *T. atrox* may have had a unique type of ecology among mammals.

## Introduction

Saber-toothed mammals were cats (family Felidae) or “cat-like” forms that have been proposed as super-ambush predators, using the enlarged blade-like upper canines to slash at the throat or belly of their prey, target areas where they would not risk breaking their dental weaponry by hitting bone (Emerson & Radinsky, 1980; Van Valkenburgh, 2007). This proposed predatory mode contrasts with that employed by extant “conical-toothed” large felids, which employ a crushing bite to the back of the skull or the nape of the neck, using a clamp-and-hold type of predatory behavior (McHenry et al., 2007; Wroe, Lowry & Antón, 2008; Figueirido, Martín-Serra & Janis, 2016; Figueirido et al., 2018).

The saber-tooth ecomorph evolved convergently several times within the placental (eutherian) mammals, both in the extant order Carnivora (in the extant family Felidae, and the extinct families Nimravidae and Barbourofelidae), and in the extinct order Oxyaenida (e.g., Van Valkenburgh, 2007). Within marsupials, the saber-tooth morphology evolved in the South American taxon *Thylacosmilus atrox* (Thylacosmilidae, Sparassodonta, Borhyaenoidea, Metatheria; Goin & Pascual, 1987). The borhyaenoid sparassodonts also included other large (∼20–200 kg), carnivorous forms such as members of the Borhyaenidae: other sparassodonts (e.g., Hathliacynidae) were in general smaller and some were apparently more omnivorous (Prevosti & Forasiepi, 2018).

Although sparassodonts should strictly be referred to as metatherians, rather than marsupials, because they lie outside of the marsupial crown group, *T. atrox* is commonly referred to as the “marsupial saber-tooth”, a term that we retain here because of its familiarity (see Goin & Pascual, 1987). The apparent craniodental similarity between the Pliocene *T. atrox* (Riggs, 1933) and the Pleistocene placental “saber-toothed tiger” *Smilodon fatalis* (Carnivora, Felidae), is often presented as a classic example of evolutionary convergence (e.g.,  Prevosti, Forasiepi & Zimicz, 2013; Harmon, 2017). Note, however, that among other differences, the jaguar-sized *T. atrox* was only around half the size (∼117 kg; Ercoli & Prevosti, 2011) of *S. fatalis*, which was lion-sized or larger (average body mass ∼245 kg; Christiansen & Harris, 2005).

Both eutherian and metatherian saber-tooths differ from conical-toothed cat-like predators in a number of ways in their craniodental anatomy, in addition to their iconically enlarged upper canines. These include an antero-posteriorly shortened and rotated braincase, a reduced coronoid process of the dentary, and a lowered glenoid fossa (Emerson & Radinsky, 1980; Antón et al., 2004; Turner et al., 2011). These osteological modifications resulted in changes in the size and orientation of the jaw adductor muscles so that they could accommodate a greater degree of stretch, allowing for a larger gape. This is further reflected in the skull by smaller and more verticalized areas of origin and insertion of the masseter and temporalis (smaller masseteric fossa and narrower width across the zygomatic arches; smaller temporal fossa and coronoid process, respectively), and in reduced moment arms for these muscles (Emerson & Radinsky, 1980). The resultant relatively weaker bite force was partially compensated for by a shorter bite force resistance arm, effecting a reduced distance between the jaw joint and the tooth row. Saber-toothed forms also possess a large mastoid process for the insertion of muscles involved in head depression (sternomastoid and cleidomastoid), interpreted as compensating for the reduced moment arm of the temporalis muscle, and allowing for powerful head deflection (Antón et al., 2004; Salesa et al., 2005).

Although all saber-tooth predators share cranial traits related to the necessity imposed by their enlarged upper canines to exert larger gapes, there were (at least) two ecological types or “ecomorphs” of saber-tooths (see Van Valkenburgh, 2007, for review): the scimitar-toothed forms (e.g., *Homotherium*) had shorter, serrated canines, and lacked a pronounced mandibular flange; the dirk-toothed forms (e,g., *Smilodon*) had longer, unserrated canines that were also narrower and more recurved, and had more pronounced cranial modifications. Scimitar-toothed saber-tooths had more cursorially-adapted postcranial skeletons than most conical-toothed felids, while dirk-toothed forms had postcrania indicative of their being powerfully built highly-specialized ambush predators (Kurtén, 1968; Martin, 1980; Meachen-Samuels, 2012; Figueirido et al., 2018; but see Martin et al., 2000). Dirk-toothed forms were the more extremely adapted forms, including the iconic *S. fatalis* of the La Brea tar pits (one of the few dirk-toothed taxa lacking a mandibular flange) and *T. atrox*.

Here we re-evaluate the text-book example of morphological convergence between *T. atrox* and *S. fatalis* (and likely all dirk-toothed placentals). We performed the following tests: (i) a critical re-examination, using correspondence analysis of discrete cranial traits, of the craniodental morphology of *T. atrox* in comparison with a diversity of extant and extinct placental cat-like predators to ascertain shared and divergent traits; (ii) simulations of craniodental performance, using Finite Element Analysis (FEA) of models of CT scanned skulls, in different predatory scenarios in both *T. atrox* and *S. fatalis* to infer differences in predatory behavior; and finally (iii) an investigation of dental microwear, using Dental Microwear Texture Analysis (DMTA), comparing *T. atrox* to several extant and extinct carnivores, including *S. fatalis*, to investigate its dietary preferences and/or the material properties of the food ingested. Our results question the interpretation of *T. atrox* having a similar type of predatory behavior to placental saber-toothed carnivores. Further, we speculate on an alternative carnivorous mode of life for *T. atrox* based on a multifaceted analysis of its morphology.

### The craniodental anatomy of *T. atrox* in comparison with placental saber-toothed carnivores

**Figure 1:**
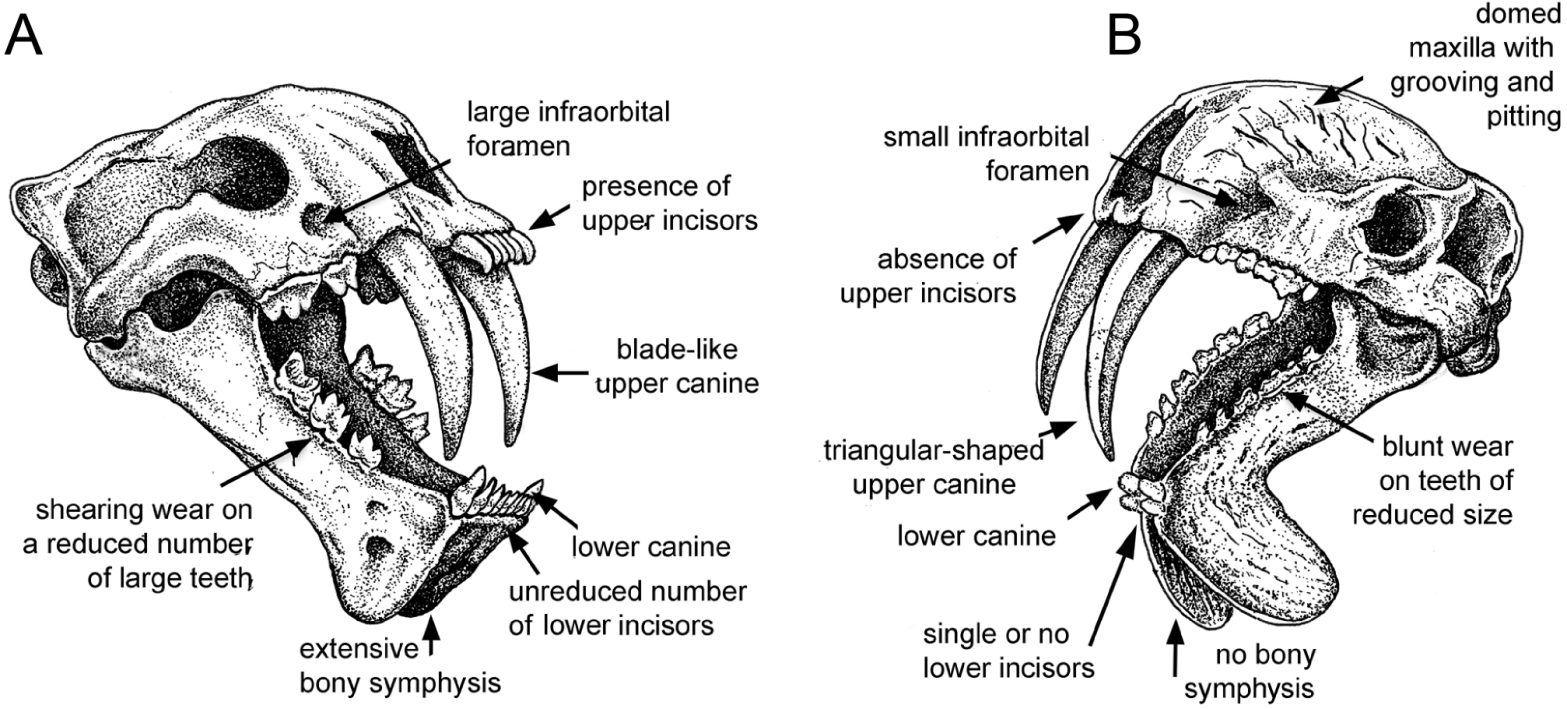
Comparison of sabre-tooth skull morphologies. (A) Skull of the felid saber–tooth Megantereon cultridens. (B) Skull of the “marsupial saber–tooth” Thylacosmilus atrox. Comparisons of saber–toothed skulls are usually portrayed in lateral view: this three-quarter view better illustrates the differences between these two animals (the splaying of the mandibles in *T. atrox* is exaggerated for effect). See text for more details. Drawing by Michael Long.

**Figure 2:**
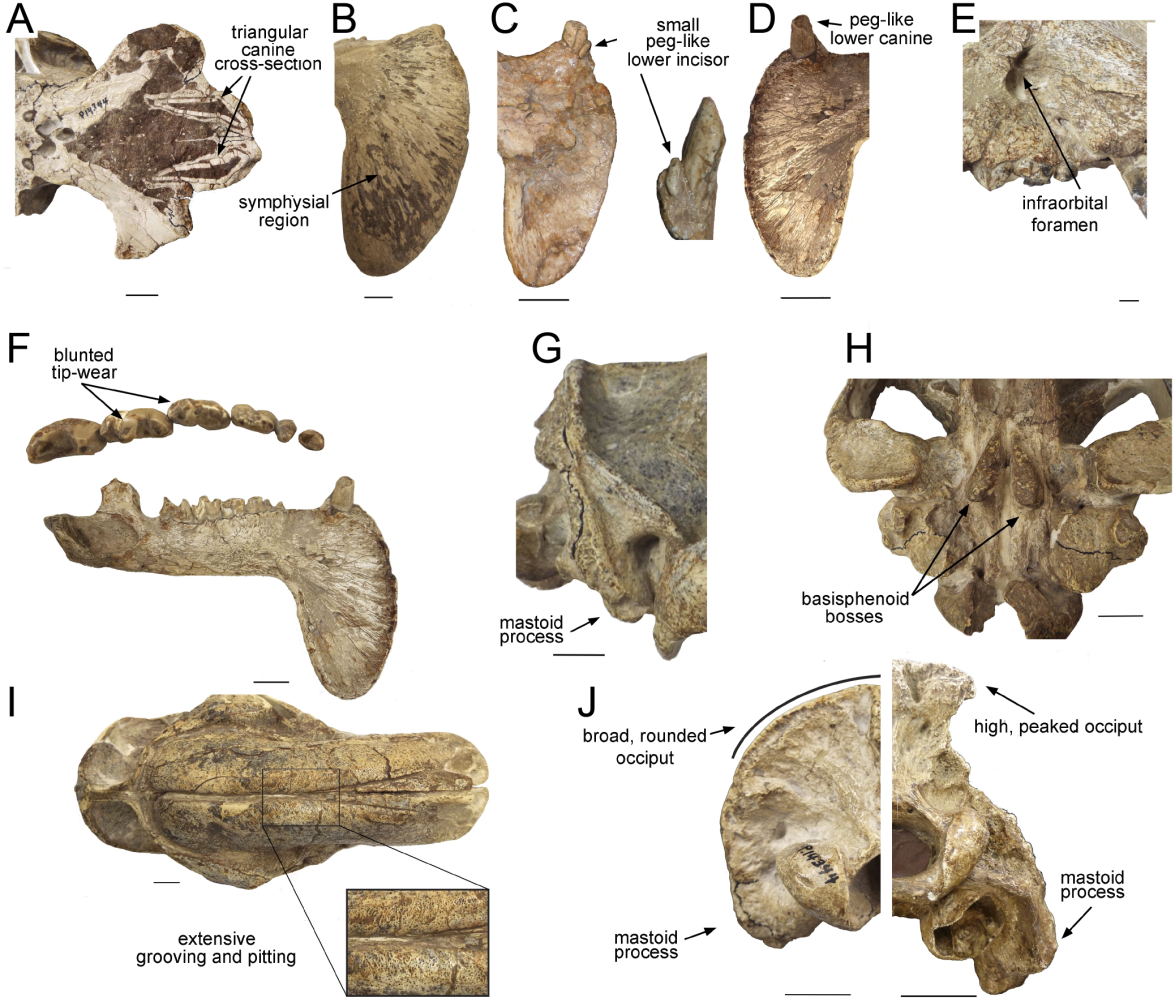
Craniodental details of *Thylacosmilus atrox*. (A) Upper canines. (B) Jaw symphysis (medial view). (C) Jaw symphysis (lateral view) showing lower incisor, plus detail of incisor. (D) Lower canines. (E) Infraorbital foramen. (F) Occlusal and lateral views of mandibular cheek teeth. (G) Mastoid process. (H) Basisphenoid bosses. (I) Maxillary bones, showing pitting and grooving. (J) Comparison of the occiput of *T. atrox* (left) and *Megantereon cultridens* (right, AMNH 113842). See text for more details. Photographs (A,B and D-J) of FMNH PP14531 (*T. atrox* holotype), P14344 (*T. atrox* paratype), and AMNH 113842 (*M. cultridens*) by Christine Janis, used here with permission, courtesy of the Department of Vertebrate Paleontology, Field Museum of Natural History, Chicago, IL, USA, and the Department of Vertebrate Paleontology, American Museum of Natural History, New York, NY, USA; C courtesy of A. Forasiepi (specimen PMM-144334). Scale bar = 3 cm.

*Thylacosmilus atrox* displays a unique combination of craniodental traits, making it unlike any placental saber-toothed carnivore (Figs. 1 and 2), differences that cannot simply be ascribed to it being a metatherian.

### Earlier thylacosmilids

Two Miocene thylacosmilids have been described and named: *Anachlysictis gracilis* (Goin, 1997) and *Patagosmilus goini* (Forasiepi & Carlini, 2010). However, both taxa, known only from partial cranial material, appear to lack the prominently developed saber-tooth characteristics of *T. atrox*. It is possible that *T. atrox* was a bizarrely specialized end-member of its family. Some researchers refer to *T. atrox* as *Achlysictis lelongi* (Ameghino, 1891), because this was the original name given to this taxon, and so should have taxonomic priority; but here we follow Goin & Pascual (1987) in retaining the commonly-used nomenclature.

### Canines

**Figure 3:**
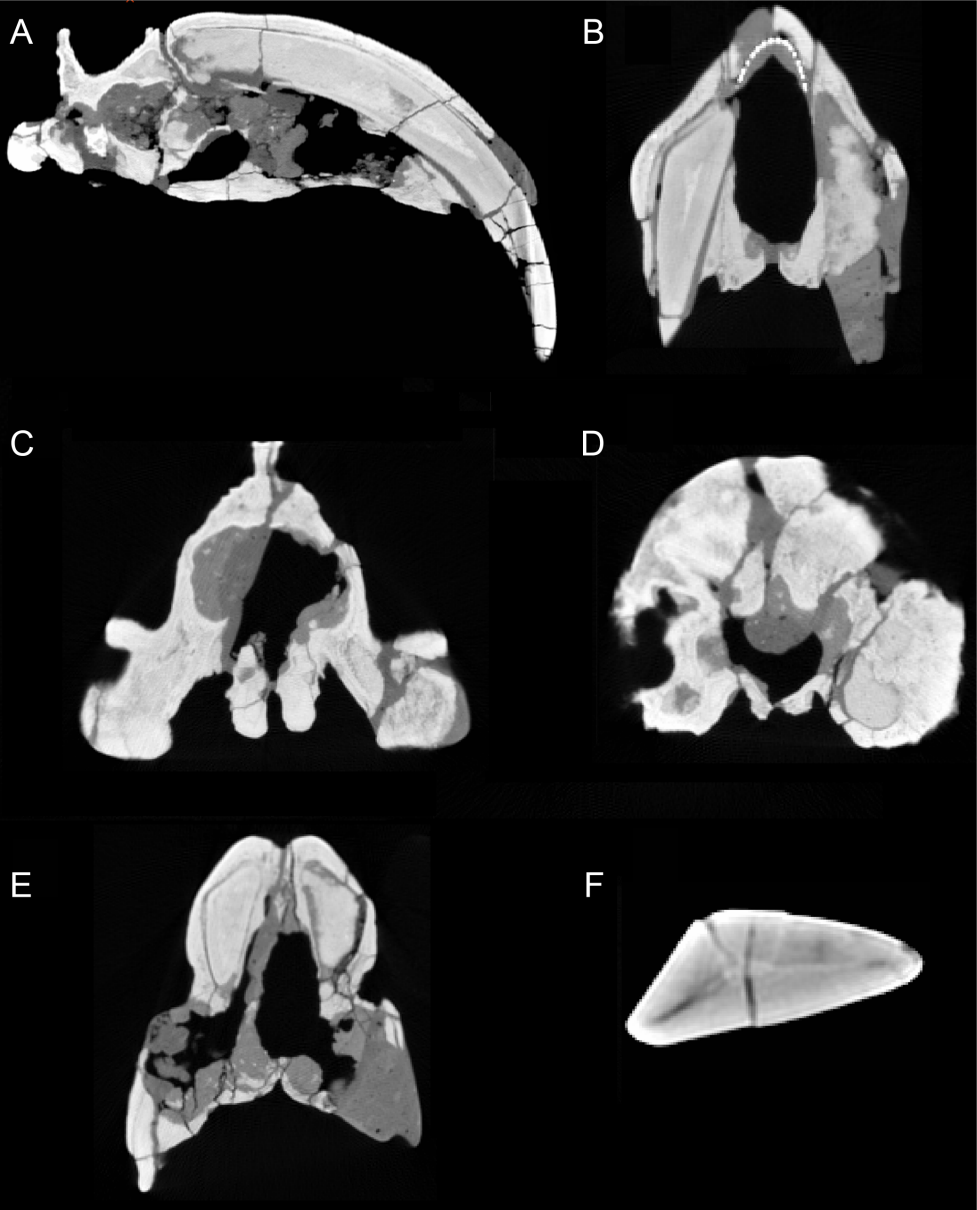
Sections through the cranium of *Thylacosmilus atrox* (CT scan of the holotype specimen, FMNH PP14531). (A) Sagittal section, showing extreme posterior extension of the canines. (B) Cross section anterior to the orbits showing the slight divergence of the canines (the dotted lines represent a metal infilling, probably inserted during the restoration of the skull). (C) Cross section posterior to the orbits showing the basisphenoid bosses. (D) Cross section through the occipital region showing the flabellate shape. (E) Cross section at around the level of the orbits showing the arched shape of the palate. (F) Cross section through the canine showing the triangular shape.

One of the most notable features of *T. atrox* is that the hypertrophied upper canines were ever-growing: Riggs (1934) describes them as having an open, trumpet-like posterior end, like the ever-growing incisors of rodents (see Fig. 3A). However, members of the Proborhyaenidae (a late Paleogene borhyaenoid family) also possessed enlarged and ever-growing upper canines (Bond & Pascual, 1983; Marshall, Case & Woodburne, 1990; Babot, Powell & Muizon, 2002). Thus this canine anatomy may be a plesiomorphic feature for *T. atrox*, rather than a specialized adaptation for an extreme saber-toothed lifestyle or evidence of some super-ability to redress canine fracture (as postulated by Therrien, 2005). The possession of ever-growing canines in some borhyaenoids may relate to the lack of canine replacement in metatherians (Churcher, 1985).

*T. atrox* has a wider overall gape (maximum angle between upper and lower jaws) than saber-toothed felids (Wroe et al., 2013), although the gape of *Barbourofelis* is of similar extent (Churcher, 1985). Additionally, the maximum angle of the gape, as measured between the tips of upper and lower canines (i.e., the effective gape), is considerably greater in *T. atrox* than in the placental saber-tooths as the upper canines of *T. atrox* are projected more anteroventrally (Churcher, 1985).

The upper canines of all saber-tooths are deeply rooted, extending to the level of the orbit (Riggs, 1934), likely reflecting anchorage to resist forces generated by stabbing (Turnbull, 1978) or by disengaging the canines from the prey (Churcher, 1985). The posterior extension of the upper canines of *T. atrox* is greater than that seen in placental saber-tooths, to a point considerably beyond the posterior border of the orbit (Riggs, 1934), and only around 45% of the total length of the canine lies outside of the alveolus (Churcher, 1985) (see Fig. 3A). This greater posterior extension of the upper canines may primarily relate to their open-rooted condition.

The upper canines of *T. atrox* also differ from those of placental saber-tooths in being subtriangular in cross-section rather than blade-like (Figs. 2A and 3F). The canines also diverge slightly (Figs. 1 and 3B) (Riggs, 1934). This divergence has been disputed (e.g., Churcher, 1985; Marshall, 1976; Turnbull, 1976; Turnbull, 1978), but it is apparent in the type specimen (Fig. 3B). Canine divergence would have impeded the proposed saber-tooth head-striking “canine-shear bite” (Akersten, 1985), in which the canines were supposedly driven into the prey in the fashion of the killing bite of extant big felids, but more extensively powered by the head deflecting muscles. Divergence of the tips of the canines would have made this action difficult or impossible for *T. atrox*, but note that Emerson & Radinsky (1980) proposed that the canine action of saber-tooths was for making a shallow wound via slashing rather than a deep bite. The upper canines of *T. atrox* are also unusual in that the enamel covering is found primarily on the lateral side, and it is extremely thin, being no more than 0.5 mm thick at any point on the tooth (Goin & Pascual, 1987; Riggs, 1934) (see Fig. 3F). Riggs (1934) noted that, while in other borhyaenoids the upper canine is frequently blunted or broken, in the specimens of *T. atrox* the canine is pointed with little signs of wear.

Other carnivorous mammals have substantial lower canines, although these are smaller in saber-tooths than in conical-toothed forms, where they are actually incisiform (Biknevicius, Van Valkenburgh & Walker, 1996). *T. atrox* has only small, peg-like lower canines (Fig. 2D), which have been proposed to be used to sharpen the upper canine via thegosis on the medial side of the tooth, causing the wearing away of the thin enamel (Goin & Pascual, 1987; Turnbull, 1978). (Note that other borhyaenoids retained substantial lower canines (Prevosti & Forasiepi, 2018)). However, Churcher (1985) disputed this potential function of the lower canine and considered that the upper canines may have been sharpened by contact with an abrasive genial pad on the mandibular flanges.

### Incisors

Placental saber-tooths (especially dirk-toothed forms) have enlarged, procumbent incisors, inferred to be used for food prehension that would otherwise be limited by the enlarged canines; the enlarged incisors may possibly also have been important in prey capture and transport (Emerson & Radinsky, 1980; Biknevicius, Van Valkenburgh & Walker, 1996). However, *T. atrox* is devoid of an incisor battery, let alone a typical saber-tooth one. Following Goin & Pascual (1987), *T. atrox* apparently lacks upper incisors entirely and retains only a single pair of small peg-like lower ones (not present in all specimens) (see Fig. 2C). Both Goin & Pascual (1987) and Churcher (1985) argued for the presence of at least one pair of upper incisors, due to the wear seen on both the lower incisors and the lower canines; however, there is no osteological evidence for their presence. Note that the complete premaxilla of *Thylacosmilus* “*lentis*” (FMNH P14474; synonymized with *T. atrox* by Marshall, 1976) lacks any evidence of incisor alveoli (Riggs, 1934).

### Postcanine teeth

**Figure 4:**
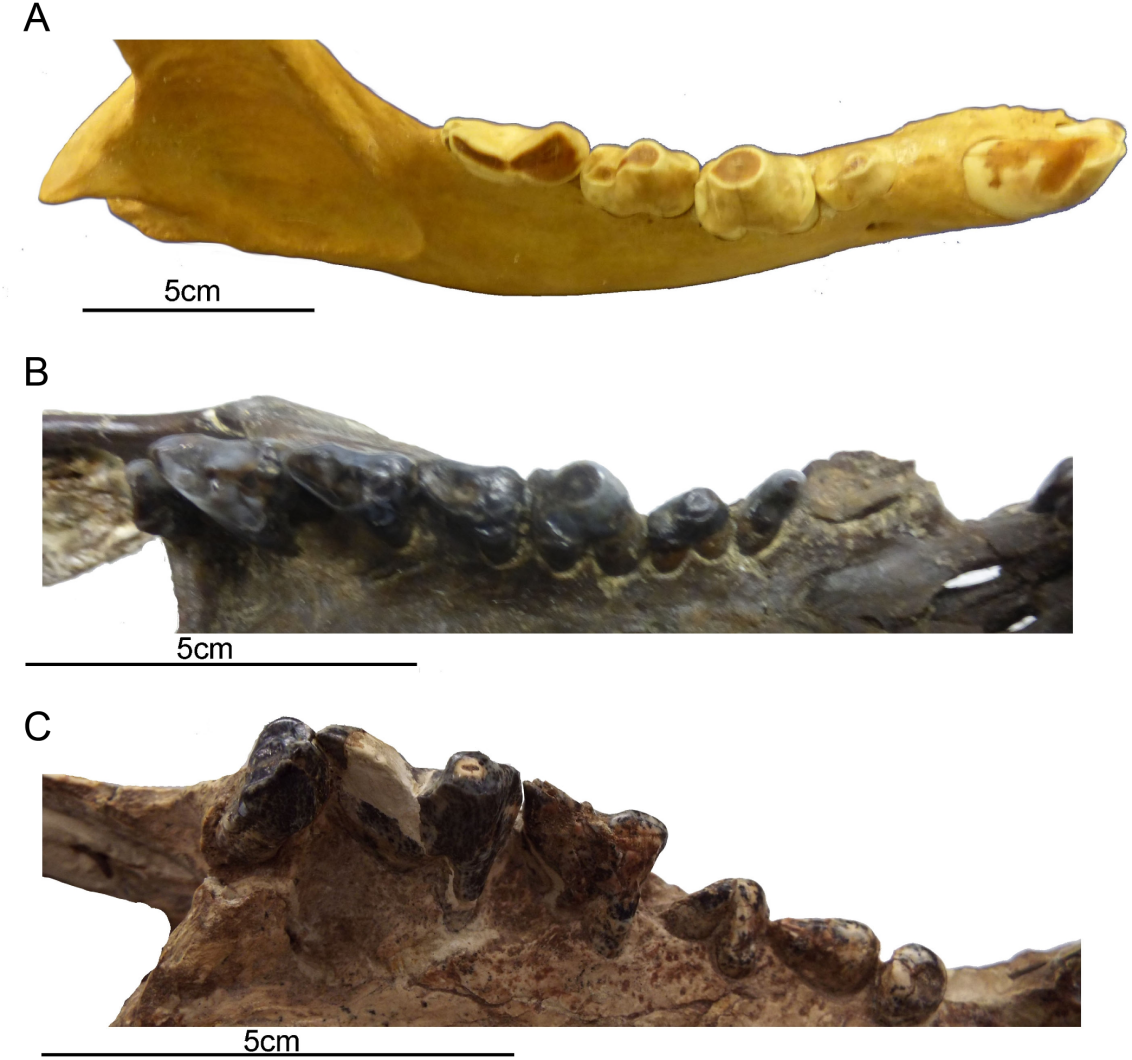
Comparison of gross dental wear in carnivorous mammals. (A) Right lower dentition of a spotted hyena (*Crocuta crocuta*, MCZ 50343, courtesy of the Department of Mammalogy, Museum of Comparative Zoology, Harvard University, MA, USA) showing a clear distinction between shearing wear on the carnassial and tip-crushing wear on the premolars. (B) Right upper dentition of a borhyaenoid sparassodont (Metatheria) (*Borhyaena tuberata*, YPM 15701, courtesy of the Department of Vertebrate Paleontology, Peabody Museum of Natural History, Yale University, New Haven, CT, USA), showing tip wear on the first molar (which resembles a hyena premolar in form) and shearing wear on the posterior molars (especially evidence on the third molar). (C) Right upper dentition of *Thylacosmilus atrox* (FMNH PP14531, holotype, courtesy of the Department of Vertebrate Paleontology, Field Museum of Natural History, Chicago, IL, USA), showing tip wear on the paracone of the third molar. Although there is some damage to the teeth, no evidence of carnivoran-like shearing wear was observed on any of the teeth of the holotype or the paratype (see lower dentition in Fig. 2). All photographs taken by Christine Janis, used here with permission from the relevant institutions.

Placental cat-like predators in the order Carnivora have a reduced postcanine dentition, focused on a single enlarged carnassial tooth in each jaw half (formed from the upper fourth premolar and the lower first molar in all carnivorans). These saber-tooths have exceptionally large carnassials, and further reduce the number and size of the non-carnassial teeth with the exception of the tooth anterior to the carnassial, which may become enlarged and somewhat carnassialized (Turnbull, 1978). Like other sparassodonts, *T. atrox* retains the full complement of cheek teeth (with the exception of the loss of the first premolars (Riggs, 1934)) and, like most other carnivorous metatherians, lacks true carnassials (Marshall, 1976; although note the presence of a single carnassial-like premolar in the diprotodontid marsupial *Thylacoleo carnifex* (Wroe, Lowry & Antón, 2008)). *T. atrox* evidences a sectorial form to the second through the fourth lower molars, and the second and third upper molars (with the third upper molar and the fourth lower molar, in particular, approaching a carnassial pair in form): the first upper molar is extremely worn, the fourth upper molar is reduced and oriented transversely, and the premolars are simple and peg-like in shape (Riggs, 1934) (see Fig. 4C). The cheek teeth appear small for the size of the animal in comparison with other borhyaenoid sparassodonts (Riggs, 1934; Turnbull, 1978).

Although the molars of *T. atrox* apparently erupted as sharp blades (see Plate I in Goin & Pascual, 1987), in the specimens that we have examined (three individuals) they exhibit blunted tip-wear when worn (Figs. 1 and 2F), rather than the shearing wear seen in the worn carnassials of most other known or presumed carnivores (including other borhyaenoid sparassodonts (Riggs, 1934)—see figures in Chapter 3 of Prevosti & Forasiepi, 2018; see also Fig. 4B). Dental tip-wear is formed by abrasion (i.e., tooth-on-food wear), usually from hard, brittle items such as bone; in contrast, the shearing along the sides of the carnassial teeth is formed by attrition (tooth-on-tooth contact), usually caused by softer, but tough and fibrous items such as flesh (Popowics & Fortelius, 1997; Evans et al., 2005). Figure 4A shows the difference between tip-crushing wear (on the posterior premolars) and shearing wear (on the first molar, or carnassial) as seen in an extant spotted hyaena (*Crocuta crocuta*), a carnivore that engages in both bone-crushing and meat eating. Interestingly, such a combination of tip crushing and shearing wear is seen on the dentition of the borhyaenid *Borhyaena tuberata* (YPM 15120) (see Fig. 4B). In contrast, in the holotype (FMNH P14531) of *T. atrox* no shearing wear is apparent, and the tip wear is especially prominent on the paracone of the third upper molar (see Fig. 4C). A greater extent of tip wear is apparent in the lower teeth in the paratype (FMNH P14344: see Fig. 2F), and also in both upper and lower cheek teeth the commercial cast (Pal-UMA 25). While such tip wear in carnivorous mammals is typically caused by bone, it may also result from eating softer, less fibrous food (such as fish) that is crushed rather than sheared (e.g., in otters (see Popowics & Fortelius, 1997) or in killer whales (see Foote et al., 2009)). As will be discussed later, the dental wear of *T. atrox* is unlikely to have been caused by bone crushing, and is a puzzling aspect of its morphology.

### Cranium

*T. atrox* resembles placental saber-tooths in aspects of the cranium that permit a wider gape, similarities that are imposed by the extremely long canines (Slater & Van Valkenburgh, 2008). *T. atrox* also has a postorbital bar, a feature only seen in barbourofelids among placental saber-tooths (but, interestingly, also seen in the marsupial carnivore *Thylacoleo*). Postorbital bars are common in ungulates and primates, but are rarely seen in carnivores: Heesy (2005) proposed that the function of this structure is to stiffen the lateral orbital wall. Emerson & Radinsky (1980) suggested that the postorbital bar in *Barbourofelis* functioned to dissipate forces resulting from a powerful bite at the enlarged carnassial, but they noted that this cannot be the explanation for this structure in *T. atrox*.

The glenoid cavity of *T. atrox* is unlike that of other sparassodonts, lacking distinct pre- and post-glenoid processes and having a shallow concavity that is wider than the mandibular condyle (Goin & Pascual, 1987); however, a shallow glenoid is the general condition in saber-tooths (Emerson & Radinsky, 1980). The glenoid is also formed entirely from the squamosal (Turnbull & Segall, 1984), in contrast to the condition in most other metatherians where the jugal makes a contribution. Goin & Pascual (1987) also noted the rather peculiar feature of palatal depressions along the inner border of the upper molars, and that the cheek tooth row is bowed out buccally. *T. atrox* lacks the usual metatherian palatal vacuities (although these are also absent in other sparassodonts) (Riggs, 1934).

Most saber-tooths have large and rounded infraorbital foramina, interpreted as reflecting a large infraorbital nerve (a branch of the maxillary nerve, cranial nerve V_2_) allowing sensory feedback from the snout for precision positioning of the upper canines (Antón, 2013); in contrast, the infraorbital foramen of *T. atrox* is relatively small and slit-like (Figs. 1 and 2E).

*T. atrox* possesses a unique anatomy of the auditory bulla (Turnbull & Segall, 1984), which is derived mainly from the mastoid and exoccipital bones, forming a large bony area at the back of the skull for muscle attachment. There is some deep contribution from the tympanic and petrosal, but there is no contribution from the alisphenoid that usually forms the bulla in other metatherians (although the anatomy in *Borhyaena* foreshadows the *T. atrox* condition). The paroccipital process is very small, located in the posteromedial portion of the expanded exoccipital. Other peculiarities of the caudal cranium, especially the nature of the tympanic cavity, are discussed by Forasiepi, MacPhee & Del Pino (2019).

A distinctive, and unique, feature of *T. atrox* is the domed nature of the skull, formed by the enlarged maxillary bones that house the upper canines (Figs. 1 and 2I). A Principal Component Analysis of the cranium of carnivorous mammals (Goswami, Milne & Wroe, 2011) showed *T. atrox* as being different from all other mammals on the second component, reflecting this extreme posterior extension of the maxillae. Some saber-toothed felids have moderately high scores on this axis, probably reflecting the enlarged maxillary housing of the upper canines. The maxillae of *T. atrox* are extensively pitted and grooved in all specimens (Figs. 1 and 2I), suggestive of some sort of soft tissue covering: very few authors have commented on this feature, although Riggs (1934, p.4) tentatively proposed a “horny covering”.

### Mandible

Like most dirk-toothed placental saber-tooths, *T. atrox* has enlarged mandibular flanges (Figs. 1 and 2F), which are relatively larger than in any placental saber-tooth (Turnbull, 1978). But while these flanges in placental saber-tooths are united by a strong bony symphysis, *T. atrox* lacks a bony union of the lower jaws, making it almost unique among mammals, although there was likely a strong ligamentous symphysial connection (Goin & Pascual, 1987; Turnbull, 1978). The flanges are heavily grooved over both lateral and medial surfaces (Figs. 2B and 2C), indicative of soft tissue coverage including nerves and blood vessels (Churcher, 1985). Churcher (1985) considered that this flexible symphysis, together with the wide and shallow jaw glenoids, would allow for lateral and antero-posterior excursions of the mandible to position the upper canines for sharpening against the lower canines or a proposed genial pad on the mandibular flanges.

The coronoid process is small, as seen in other saber-tooths (Emerson & Radinsky, 1980) (see Fig. 2F). The mandibular body is extremely slender, and the mandibular condyle is small in proportion to the glenoid (Goin & Pascual, 1987). The analyses of Echarri et al. (2017) classified the shape of the mandible of *T. atrox* as that of an hypercarnivorous mammal; but note that their graphs show its mandibular shape falls outside of the range of extant mammals in most analyses (probably because of the mandibular flanges), and *T. atrox* does not cluster close to any other sparassodont taxon.

### Jaw Musculature

Goin & Pascual (1987) describe the mandible as having a very low ramus, a small masseteric fossa, and a poorly-inflected and posteriorly-angled angular process, features which all imply relatively small volumes of the jaw adductor muscles. The relative proportions of the jaw adductor muscles of *T. atrox* are similar to those seen in other carnivorous mammals (Turnbull, 1976), but the volume of musculature relative to the size of the animal was low in comparison to a saber-tooth such as *S. fatalis* (Wroe et al., 2013).

Wroe et al. (2013) applied computational modelling to the masticatory musculature of *T. atrox* in comparison with a saber-toothed felid (*S. fatalis*) and an extant large conical-toothed felid (*Panthera pardus*), showing that the bite force of *T. atrox* was extremely weak in comparison with the other cat-like carnivores, with the implication of a minor role of the jaw-adductors in the killing bite.

### Occiput and neck

*T. atrox* resembles some of the most specialized dirk-toothed placental saber-tooths in having occipital condyles that strongly protrude from the posterior aspect of the cranium (Emerson & Radinsky, 1980; Goin & Pascual, 1987). This has been interpreted as a modification for maximizing the capacity for head dorsiflexion, a cranial action that may be essential for an animal with extremely long upper canines, irrespective of how those canines were employed. However, *T. atrox* differs from placental saber-tooths such as *S. fatalis*, and as best as we can determine also differs from other borhyaenoids (see e.g., figures in Chapter 3 of Prevosti & Forasiepi, 2018), in the relatively dorsal position of the occipital condyles. This position would indicate a more horizontal orientation of the head on the neck; this would not be advantageous for flexing the head ventrally, as would be necessary for a predatory head strike, but might be advantageous for bracing the head on the neck while pulling back with the canines (see later discussion).

Both *T. atrox* and placental saber-tooths have a cranial and cervical anatomy indicative of powerful musculature to move and stabilize the head on the neck, but the anatomies are somewhat different, indicative of different functional morphology and hence different behavior. The occiput of *T. atrox* is large and broad (like that of other borhyaenoids), with pronounced rugosities for muscle attachment, indicative of the insertion of powerful head elevators (Argot, 2004; Riggs, 1934; Turnbull, 1976). The skull of *Borhyaena tuberata* (YPM 15120) shows a similarly shaped dorsal portion of the occiput, but it lacks the ventral expansion of the mastoid/exoccipital area seen in *T. atrox* (Figs. 2J and 3D). However, the occiput of placental saber-tooths is very different in shape (Fig. 2J); it is dorsally narrow and markedly peaked (or more moderately peaked in extant large cats). The peaked occiput of placental saber-tooths would provide an advantageous angle for the action of head elevators (especially the semispinalis capitis and rectus capitis dorsalis minor) for the extent of head elevation necessary for head-striking predatory behavior (Matthew, 1910).

The atlas of *T. atrox* has large, posteriorly-directed transverse processes (Riggs, 1934) (an anatomy also seen in placental saber-tooths, but to a greater extent in *T. atrox,* at least in comparison with *S. fatalis*; Argot, 2004), indicative of large obliquus capitis muscles. The oblique capitus cranialis (= superior) muscles run between the dorsal portion of the transverse processes of the atlas and the occiput, acting to flex or incline the head. The oblique capitus caudalis (= inferior) muscles run between the dorsal portion of the transverse processes of the atlas and spinal process of the axis, stabilizing the atlanto-axial joint or rotating the head.

Dirk-toothed placental saber-tooths have large, ventrally-projecting mastoid processes, indicative of powerful sternomastoid muscles for the depression of the head and considered as enabling the use of these muscles to depress the head in a powerful predatory strike (Matthew, 1910; Emerson & Radinsky, 1980). Antón et al. (2004) took a different view of this anatomy, considering that the ventrally-projecting mastoid processes reflect large and ventrally-extended obliquus capitis cranialis muscles, and that it is these muscles that are primarily implicated in saber-tooth predatory head strike behavior. Although the mastoid processes of *T. atrox* are robust (Riggs, 1934; Turnbull, 1976), they do not project ventrally below the occiput to any great extent (Figs. 2G, 2J and 3D), and thus would not provide the type of leverage for either sternomastoid or obliquus capitis cranialis muscles proposed to be important for a saber-tooth predatory head strike. The biomechanical analysis of Wroe et al. (2013) reconstructed *T. atrox* as having much smaller sternomastoid and obliquus capitis cranialis muscles than *S. fatalis*. Their study also shows that with forces generated by head depression the force at the canines of *T. atrox* would be only about half that of *S. fatalis*.

However, *T. atrox* possesses large basisphenoid bosses on the underside of the skull (Figs. 2H and 3C), which have been proposed as the origin of a different set of powerful head depressors; i.e., the longus capitis (Riggs, 1934) (= *M. rectus capitis ventralis major* of Turnbull & Segall, 1984). In most mammals these muscles originate from the transverse processes of the third through sixth cervical vertebrae and insert onto the basioccipital-basisphenoid suture. *T. atrox* also evidences strong ventral projections for the origin of these muscles on the cervical vertebrae (Riggs, 1934); note that these projections are also present in other borhyaenoids, but absent in *S. fatalis* (Argot, 2004). However, Emerson & Radinsky (1980) noted that the longus capitis muscles would have relatively poor leverage for head flexion.

Basisphenoid bosses like those seen in *T. atrox* are seen in bovids (termed “muscular tubercles”), where they serve as the insertion point of the longis capitis and also as areas of origin for portions of the sternomastoid and cleidomastoid muscles (Brudas & Hagel, 2011). However, in other domestic mammals the sternomastoid and cleidomastod muscles originate solely from the mastoid processes (Nickel et al., 1986). It is possible that the enlarged basisphenoid bosses of *T. atrox* signify a bovid-like condition of the origin (in part) of the sterno- and cleidomastoid muscles, or they may simply indicate a large longis capitis.

In summary, it appears that although *T. atrox* evidently had powerful musculature running between the head and the neck, these muscles were positioned along the axis of the neck, suitable for stabilizing the head and resisting torsion and rotation, but not well-positioned for powerful head elevation and depression. *T. atrox* lacks the anatomical features of placental saber-tooths that indicate muscles with sufficient leverage to elevate the head to a pronounced extent (i.e., the peaked occiput) or to depress the head forcefully and rapidly (i.e., the elongated mastoid processes). The anatomy of *T. atrox* appears indicative of strong musculature to stabilize the head on the neck, which may have aided the proposed pull-back action described later, rather than to effect the type of predatory head strike proposed for placental saber-tooths.

## Materials and Methods

### Correspondence analysis

This analysis provides a graphical assessment of morphological variability (see Werdelin & Lewis, 2013). Here we coded discrete craniodental morphological traits in a sample of “cat-like” predators (Table 1). For the saber-toothed forms (including both scimitar-and dirk-toothed morphs) we included machairodontine felids (Carnivora, Felidae, Machairodontinae), and both families of “false saber-tooths”, nimravids and barbourofelids (Carnivora; Nimravidae and Barbourofelidae, respectively). Conical-toothed forms included a diversity of cats (Carnivora, Felidae, Felinae), including medium-sized (e.g., *Lynx lynx*) and large (e.g., *Panthera leo*) forms, and in addition the cat-like *Cryptoprocta ferox*, the fossa (Carnivora, Eupleridae). Details of the specimens studied are provided in Table 2. Data on *T. atrox* came from study of the original holotype (FMNH P14531), and the paratype (FMNH P14344), from the Huayquerian, Province of Catamarca (Argentina) (Riggs, 1934) and of a commercial cast (Pal-UMA 25) housed at the paleontological collections of the University of Málaga (Spain) (see the end of this section for a list of institutional abbreviations).

**Figure 5:**
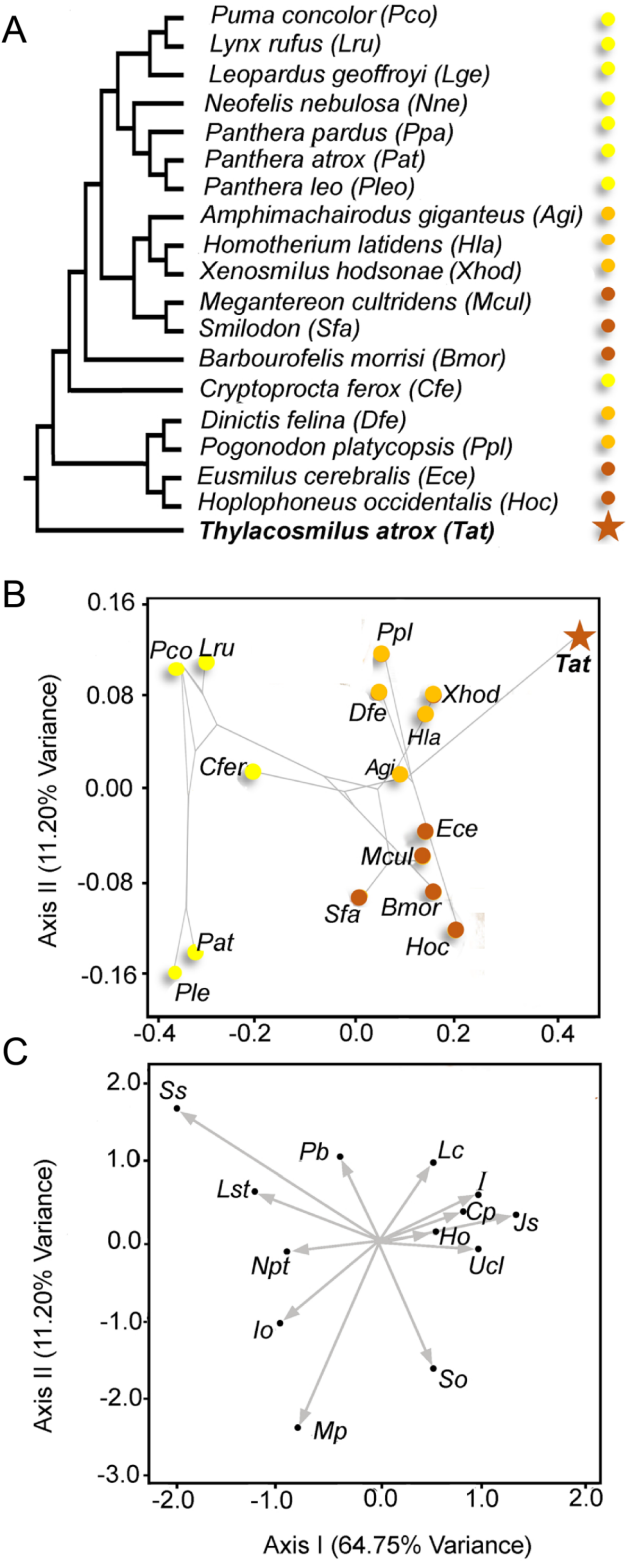
Multivariate analysis of discrete morphological traits of saber-tooths and conincal-toothed carnivores. (A) Phylogeny employed. Yellow, conical-toothed carnivores (extant felids and euplerids); orange, scimitar-toothed saber-tooths; red, dirk-toothed saber-tooths. (B) Phylomorphospace depicted from the scores of the taxa taken from the first two axes of a Canonical Correspondence analysis (See Table 1). (C) Representation of the variable loadings on both multivariate axes (Table 1 shows the abbreviations of the morphological traits). Table 2 shows the details of the taxa studied.

We assembled a phylogenetic consensus tree to assess phylogenetic patterning with Mesquite (Maddison & Maddison, 2011) (Fig. 5A). For the phylogeny of Machairodontinae, we used the tree of Piras et al. (2013) and for the Felinae we used Johnson et al. (2006). We considered Nimravidae to be basal to the other Feliformia (Neff, 1983; Peigné, 2003).

We computed a Correspondence Analysis using Past (Hammer, Harper & Ryan, 2001) and superimposed this phylogeny on the first two axes to represent a phylomorphospace (Fig. 5B) using the PDAP module (Garland et al., 2002) of Mesquite (Maddison & Maddison, 2011).

The traits selected represent features known to distinguish saber-tooths from other carnivores (Emerson & Radinsky, 1980; Van Valkenburgh, 2007; Slater & Van Valkenburgh, 2008; Wroe et al., 2013). Because we specifically considered saber-tooth specializations, we did not include other types of carnivorous mammals, and we avoided many of the features unique to *T. atrox* (e.g., domed maxillae) so as not to bias the analysis in favor of the distinctiveness of this animal (although we did include a few unique features: greatly reduced or absent incisors, triangular-shaped upper canine, and ligamentous jaw symphysis).

**Table 1:** Discrete morphological traits used in the correspondence analysis.

	**Npt**	**Ucl**	**Pb**	**I**	**Ho**	**So**	**Cp**	**Mp**	**Js**	**Lc**	**Io**	**Lst**	**Ss**
*Thylacosmilus atrox*†	1	4	2	4	3	3	3	1	5	3	1	1	1
*Barbourofelis morrisi*†	3	3	2	3	3	4	3	2	4	2	3	2	1
*Hoplophoneus occidentalis*†	2	3	1	3	3	4	3	2	4	2	3	2	1
*Eusmilus cerebralis*†	3	3	1	3	3	4	3	2	4	2	3	2	2
*Dinictis* sp.†	1	2	1	2	3	2	2	1	3	2	3	2	2
*Pogonodon platycopsis*†	2	2	1	2	3	2	2	1	3	2	2	2	2
*Smilodon californicus*†	3	3	1	2	3	2	2	2	2	2	3	2	1
*Megantereon cultridens*†	2	3	1	2	3	2	2	2	4	2	3	2	1
*Xenosmilus hodsonae*†	2	2	1	2	3	2	2	1	3	2	3	2	1
*Amphimachairodus giganteus*†	3	2	1	3	3	2	3	1	3	2	2	2	1
*Homotherium crenatidens*†	2	2	1	2	2	2	3	1	3	2	2	2	1
*Neofelis nebulosa*	2	1	1	1	1	1	1	1	1	1	2	2	2
*Panthera pardus*	2	1	1	1	1	1	1	1	1	1	2	2	2
*Panthera leo*	2	1	1	1	1	2	1	2	1	1	3	2	2
*Panthera atrox*†	2	1	1	1	2	2	1	2	1	1	3	2	2
*Felis concolor*	2	1	1	1	2	1	1	1	1	1	2	2	2
*Oncofelis geoffroyi*	2	1	1	1	1	1	1	1	1	1	2	2	2
*Lynx rufus*	2	1	1	1	1	1	1	1	1	1	2	2	2
*Cryptoprocta ferox*	1	1	1	1	1	1	1	1	1	1	2	2	1

**Notes.**

Npt, number of postcanine teeth: (1) >3 teeth; (2) 3 teeth; (3) <3 teeth). Ucl, Upper canine length: (1) Short, conical; (2) Scimitar-blade; (3) Dirk-blade; (4) Dirk-triangular. Pb, post-orbital bar: (1) Absent; (2) Present. I, incisors: (1) Small, straight; (2) Large, straight; (3) Large, protruding; (4) None. Ho, height of the occiput: (1) Low; (2) Medium; (3) High. So, shape of the occiput: (1) Truncate (low); (2) Deltoid (moderately peaked, triangular); (3) Flabellate (broad, fan-shaped); (4) Hastate (highly peaked, spear-shaped). Cp, coronoid process: (1) Large, projecting backwards; (2) medium-sized, projecting backwards; (3) Small, projecting dorsally. Mp, mastoid process. (1) Does not project far ventrally below occiput; (2) Projects far ventrally below level of occiput. Js, jaw symphysis: (1) Short, narrow; (2) Short, broad; (3) Medium-length, broad. (4) Long, broad; (5) Long, ligamentous. Lc, lower canine: (1) Large; (2) Small; (3) Peg-like. Io, Infraorbital foramen: (1) Small, slit-shaped; (2) Small, round; (3) Large, round. Lst, Largest sectorial tooth: (1) Blunt tip wear; (2) Sharp shearing wear. Ss, Skull shape: (1) Rounded; (2) Flat.

### Finite element analysis (FEA)

For the biomechanical analysis, digital skull models of *Thylacosmilus atrox* and *Smilodon fatalis* were generated on the basis of computed tomography (CT) scans. The skull of *T. atrox* (FMNH P14531) was scanned at O’Bleness Memorial Hospital in Athens, OH, USA, using a General Electric LightSpeed Ultra MultiSlice CT scanner at 120 kV and 200 mA with Extended Hounsfield engaged and bone-reconstruction algorithm. Data were resampled to consist of 432 DICOM images with an isotropic resolution of 300 µm. The skull of *S. fatalis* (LACMRLP R37376) was scanned at the University of Texas High-Resolution X-ray Computed Tomography Facility using a Zeiss microXCT 400 scanner at 420 kV and 180 mA and is available on the UT Digital Morphology website (http://www.digimorph.org). Data were resampled to consist of 629 TIFF images (1,024 × 1,024 pixel) with a resolution of 210 µm (X, Y) × 500 µm (Z).

For model construction, data sets were imported into Avizo (version 9.0, Thermo Fisher Scientific) and bones and teeth were segmented using a combination of automatic thresholding and manual segmentation using Avizo’s segmentation editor. Taphonomic artefacts, such as cracks and fractures, unilaterally missing elements and slight deformation were removed during the segmentation following protocols outlined in Lautenschlager (2016). Models were exported as HMASCII files for subsequent processing.

In addition to the original model of *T. atrox*, two further hypothetical models were created to test the biomechanical contribution of specific osteological features: (i) a model of *T. atrox* with the postorbital bar digitally removed, and (ii) a model with the root of the canine tooth reduced in length of that of *S. fatalis*. The 3D models were imported into Hypermesh (version 11, Altair Engineering) for the generation of solid meshes (consisting of approximately 1,000,000 tetrahedral elements per model) and the setting of boundary conditions. The skull models were scaled to the same surface area to allow comparisons of form and function independent of size and material properties for bone and teeth were assigned in Hypermesh based on published values in comparable studies on mammalian carnivores (bone: *E* = 13.7 GPa, *ν* = 0.30, teeth: *E* = 38.6.0 GPa, *ν* = 0.4) (Figueirido et al., 2018). All materials were treated as isotropic and homogeneous. Bone and teeth were assigned single material properties, as some of the CT data did not allow differentiating individual components (e.g., cortical vs. trabecular bone, dentine vs. enamel).

**Table 2:** Specimens studied for the correspondence analysis of morphological variables.

Taxon	Canine	Order	Family (Subfamily)	Specimen No
*Thylacosmilus atrox*	Dirk	Sparassodonta	Thylacosmilidae	FMNH P14531, P14344
*Barbourofelis morrisi*	Dirk	Carnivora	Barbourofelidae	UNSM 76000
*Hoplophoneus occidentalis*	Dirk	Carnivora	Nimravidae	AMNH 102394
*Eusmilus cerebralis*	Dirk	Carnivora	Nimravidae	JODA 7047
*Dinictis* sp.	Scimitar	Carnivora	Nimravidae	UNSM 25512
*Pogonodon platycopsis*	Scimitar	Carnivora	Nimravidae	AMNH 6938
*Smilodon californicus*	Dirk	Carnivora	Felidae (Machairodontidae)	AMNH 14349
*Megantereon cultridens*	Dirk	Carnivora	Felidae (Machairodontidae)	AMNH 105446
*Xenosmilus hodsonae*	Scimitar	Carnivora	Felidae (Machairodontidae)	Pal-UMA 23 (cast)
*Amphimachairodus giganteus*	Scimitar	Carnivora	Felidae (Machairodontidae)	AMNH 144433
*Homotherium crenatidens*	Scimitar	Carnivora	Felidae (Machairodontidae)	Pal-UMA 59 (cast)
*Neofelis nebulosa*	Conical	Carnivora	Felidae (Felinae)	UNSM ZM-16951
*Panthera pardus*	Conical	Carnivora	Felidae (Felinae)	UNSM ZM-25017
*Panthera leo*	Conical	Carnivora	Felidae (Felinae)	UNSM ZM-5150
*Felis concolor*	Conical	Carnivora	Felidae (Felinae)	UNSM ZM-30745
*Oncifelis geoffroyi*	Conical	Carnivora	Felidae (Felinae)	UNSM ZM-20845
*Lynx rufus*	Conical	Carnivora	Felidae (Felinae)	UNSM ZM-14701
*Cryptoprocta ferox*	Conical	Carnivora	Eupleridae	UNSM ZM-30748

We tested here three theoretically possible functional scenarios following Figueirido et al. (2018): (i) stabbing prey using both canine teeth with a dorsally directed extrinsic force of 500 N applied to the tips of both canines; (ii) pulling the head posteriorly with both canine teeth embedded in the prey and an anteriorly directed extrinsic force of 500 N distributed (on five nodes) over the posterior edge of the canines; and (iii) shaking the head laterally while holding prey with both canine teeth and an extrinsic force of 500 N applied to the left side of both canines. The extrinsic force (total of 1,000 N for each scenario) was selected based on reported magnitudes for neck-muscle-driven bite force (McHenry et al., 2007). This scenario was modelled to rule out the possibility that the skull of *T. atrox* was well-equipped to support extrinsic head-shaking loads derived from the “clamp-and-hold” technique that large cats deploy today to kill their prey, following McHenry et al. (2007) and Figueirido et al. (2018).

For all scenarios, constraints were placed on the articular surface of the squamosal (five nodes on each side), as well as the occipital condyles (ten nodes) to restrain the model from movement in *x*-, *y*- and *z*-directions. Only extrinsic scenarios were tested to limit the comparison of biomechanical function to skull and tooth shape and to avoid effects based on differences in size and orientation of the jaw adductor musculature.

All models were imported into Abaqus (version 6.141, Simulia) for analysis and post-processing. Biomechanical performance for the FE models was assessed via contour plots of Von Mises, compressive and tensile stress distributions, deformation magnitudes and average Von Mises stress values per element. Differences between models and simulated scenarios were tested statistically using a variation of the interval method (Marcé-Nogué et al., 2017), subdividing stress magnitudes into 50 different equal ranges. These were then subjected to an analysis of variance (ANOVA) to test for significant differences (Table 3).

### Dental microwear texture analysis

The teeth of *Thylacosmilus atrox* specimens FMNH P14344 and FMNH P14531 were thoroughly cleaned with acetone and cotton swabs prior to subsequent molding and casting with polyvinylsiloxane dental impression material and Epotek 301 epoxy resin and hardener, respectively. Teeth were subsequently scanned and analyzed using a Sensofar PLu neox optical profiler at Vanderbilt University (all primary data are included in Table 4) in three dimensions with 100x magnification (using a lens with a 0.73 numerical aperture) and white-LED light. In contrast to the single pair of carnassials used for shearing in carnivorans, carnivorous marsupials (e.g., *Dasyurus*, *Sarcophilus*, *Thylacinus*) all have multiple “carnassial-like” teeth. Thus, due to the limited number of individual *T. atrox* specimens and the inferred similarity in tooth function based on similar morphology of the molars, all upper and lower molars with ante-mortem microwear were analyzed (Table 4).

**Table 3:** Qunatitiative results of the finite element analyses. Average stress magnitudes (per element averages) for each species and feeding scenarios and ANOVA results. The top right section shows p-values for Von Mises stress, the bottom left section shows *p*-values for tensile and compressive stresses. Significant differences highlighted in bold.

**Average stress magnitudes (per element average)**						
	*Thylacosmilus*			*Smilodon*		
	STAB	PULL	SHAKE	STAB	PULL	SHAKE
Von Mises	1.475	1.550333	0.76906	0.985	2.101931	1.402
Tensile	2.593	1.639	0.403	0.783	1.82	1.392
Compressive	−1.392	−1.193	−0.562	−0.928	−1.028	−1.294

**Comparison of stress magnitudes (per model, based on ANOVA)**							
		*Thylacosmilus*			*Smilodon*		
		STAB	PULL	SHAKE	STAB	PULL	SHAKE
*Thylacosmilus*	STAB		**0.001285**	0.414	0.1189	**0.001286**	**0.01404**
	PULL	**0.01278**		**3.73E−02**	**0.000152**	0.1043	**0.000133**
	SHAKE	**0.03618**	**6.54E−05**		0.7027	**5.71E−03**	**0.0112**
*Smilodon*	STAB	0.8513	**0.00959**	**0.03801**		**0.001038**	**0.01782**
	PULL	0.1237	0.2551	**0.001283**	0.1053		**7.57E−05**
	SHAKE	**0.001775**	**2.71E−06**	**0.039968**	**0.000799**	**2.51E−05**	

**Table 4:** Dental microwear attribute data for all *Thylacosmilus* teeth examined.

**Museum**	**Catalogue Number**	**Tooth position**	***Asfc***	***epLsar***
FMNH	P14344	lm2	1.659	0.0018
FMNH	P14344	lm3	1.566	0.0025
FMNH	P14344	lm4	1.080	0.0028
FMNH	P14531	LM2	1.112	0.0013
FMNH	P14531	RM1	1.358	0.0023
FMNH	P14531	RM2	1.287	0.0026
FMNH	P14531	RM3	1.571	0.0024
FMNH	P14531	RM4	1.470	0.0027

**Notes.**

lm lower left molar 2–4 LM2 upper left second molar RM upper right molars 1–4

All specimens were scanned in three dimensions in 9 areas (in a 3x3 grid), subsequently stitched together, leveled, and then subdivided into four adjacent areas of equal size (102 × 138 µm^2^) for a total sampled area of 204 × 276 µm^2^, identical sized areas as previously published DMTA data (with a sampling resolution of 36.33 data points per 1 µm2 and a step height of 0.2 µm: DeSantis, 2018; DeSantis et al., 2012; DeSantis et al., 2017; DeSantis et al., 2019). The measured neighbor algorithm was applied to all areas on the scan where no data were collected, this is typically due to steep surfaces and approximately <2% of a given surface, and resulting surface files (.sur) were created (this was necessary for consistency in DMTA attribute values due to discrepancies in how surface profilers and subsequent software treat missing data; see Arman et al., 2016). Surface files were subsequently analyzed via scale-sensitive fractal analysis using ToothFrax software (http://www.surfract.com) to characterize tooth surfaces according to the variables of anisotropy (*epLsar*) and complexity (*Asfc*). Complexity is the change in surface roughness with scale and used to distinguish taxa that consume hard, brittle foods (such as bone in carnivorous animals) from those that eat softer ones (e.g., Schubert, Ungar & DeSantis, 2010; DeSantis & Patterson, 2017; Stynder et al., 2019). Anisotropy is the degree to which surfaces show a preferred orientation, such as the dominance of parallel striations having more anisotropic surfaces (as can occur in those eating primarily tough foods—including flesh (Schubert, Ungar & DeSantis, 2010)). All data used for comparison (i.e., DeSantis, 2018; DeSantis & Patterson, 2017; DeSantis et al., 2012; DeSantis et al., 2017; DeSantis et al., 2019) were scanned on either the identical microscope or on a white-light confocal microscope at the University of Arkansas; these confocal microscopes yield DMTA data statistically indistinguishable from one another (see Table 5 in Arman et al., 2016).

All DMTA attribute values were compared to extant and extinct taxa using non-parametric statistics (Kruskal-Wallis tests and Dunn’s procedure absent of the Bonferroni correction, as the Bonferroni correction increases the probability of Type II errors; Cabin & Mitchell, 2000; Dunn, 1964; Nakagawa, 2004).

### Institutional abbreviations

These abbreviations represent the institutions cited in the text, tables, and figure captions.

AMNH, American Museum of Natural History (New York, NY); FMNH, Field Museum of Natural History (Chicago, IL, USA); JODA, John Day Fossil Beds Museum (Kimberly, OR, USA); LACMHC, Los Angeles County Museum of Natural History Hancock Collection (Los Angeles, CA); LACMRLP, Los Angeles County Museum of Natural History Rancho La Brea Project (Los Angeles, CA, collected after the Hancock Collection specimens); USNM, National Museum of Natural History (Smithsonian Institution, Washington, DC, USA); Pal-UMA, paleontological collections, University of Málaga (Málaga, Spain); PMM, Museo Municipal de Mar de Plata “Lorenzo Scaglia”, Argentina; UNSM, University of Nebraska State Museum (Lincoln, NB, USA); YPM, Yale Peabody Museum (New Haven, CT, USA).

## Results

### Correspondence analysis

Figure 5B shows the scores of the specimens in the morphospace depicted by the first two axes (explaining >75% of the variance). The first axis separates the extant conical-toothed carnivores (negative values) from the extinct saber-toothed forms (central values), while *Thylacosmilus atrox* is separated from all other carnivores with an extreme positive value. Figure 5C shows the direction and strength of the variable loadings responsible for the distribution of the taxa in Fig. 5B, showing that placental saber-tooths are characterized by many craniodental features in addition to large upper canines: for example, high (Ho) and peaked (So) occiputs, short coronoid processes (Cp), large incisors (I), large mastoid processes (Mp), and a broad, elongated jaw symphysis (Jo).

The second axis separates saber-tooths with longer canines (dirk-toothed, negative values) from those with relatively shorter canines (scimitar-toothed, positive values). Although *T. atrox* resembles scimitar-toothed forms in having positive values on this axis (unlike other dirk-toothed forms), note that it does not cluster with them on the first axis, but occupies a unique position with more positive scores than any other taxon. The position of *T. atrox* on the second axis may be determined by the small infraorbital foramen, the large number of postcanine teeth, and the lack of large incisors (see Fig. 5C), features in which it is divergent from dirk-tooths rather than being similar to scimitar-tooths. Among conical-toothed forms, the second axis separates large species (i.e., *Panthera leo* and *P. atrox*) from smaller ones. This pattern agrees with results obtained by other researchers (e.g., Slater & Van Valkenburgh, 2008): while skull shape in saber-toothed forms is mainly governed by the length of the canine, in conical-toothed forms it is mainly influenced by size.

**Figure 6:**
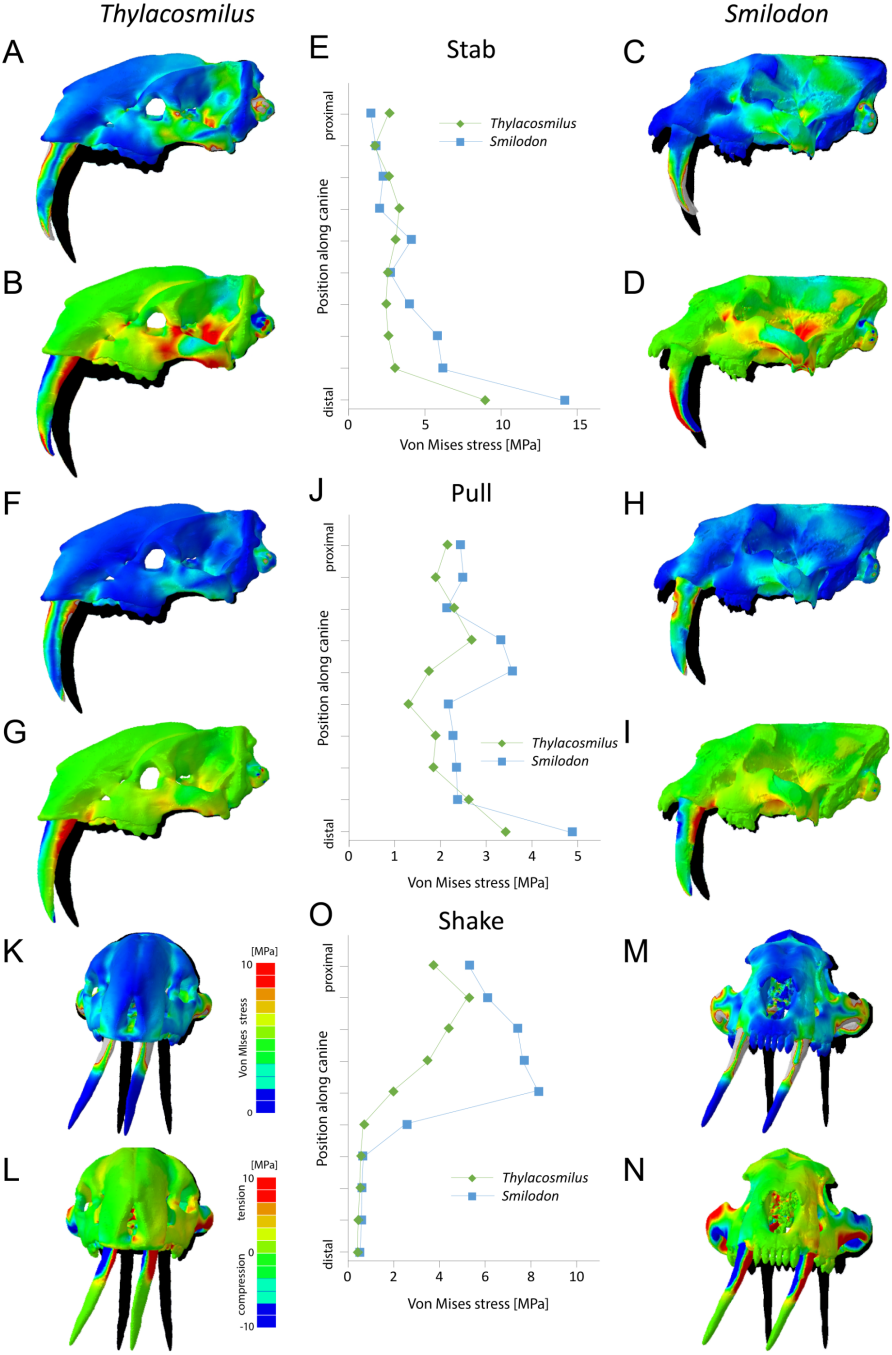
Finite element analysis results for different functional scenarios for *Thylacosmilus atrox* (FMNH P14531) and *Smilodon fatalis* (LACMRLP R37376). For each scenario deformed skull models with von Mises stress contour plots (A, C, F, H, K, M) and tensile/compressive stress plots (B, D, G, I, L, N) are superimposed on the original, undeformed shape shown in black. Deformation is exaggerated by a factor of 100. Quantitative von Mises stress magnitudes are plotted for equidistant points along the centre of the canine for each taxon (E, J, O).

### Finite element analysis

Results from the biomechanical analyses reveal distinct differences between the two studied species for the tested functional scenarios (see Fig. 6, Table 3). When considered as a whole, the cranial structure of *T. atrox* experiences lower von Mises stress magnitudes during the pull-back and lateral shake scenarios than *S. fatalis*. In *S. fatalis*, stress hotspots are centered on the nasal region, the zygomatic arches and the skull roof in these two scenarios. In contrast, the skull of *T. atrox* experiences increased stress magnitudes during simulated canine-stabbing, in particular at the posterior skull region, in comparison to *S. fatalis*. Compressive and tensile stresses show a similar pattern, with the skull of *T. atrox* experiencing high compressive stresses posterior to the orbit and at the lateral braincase wall during stabbing; these stresses are less pronounced in *S. fatalis*. The analysis of variance (ANOVA) of the tested scenarios confirms these results quantitatively (see Table 3). In particular, there is a statistically significant difference between *T*. *atrox* and *S*. *fatalis* for the lateral shake scenario.

The biomechanical differences are further shown qualitatively by the degree of deformation experienced by the cranium in the different scenarios. During simulated stabbing, the skull of *T. atrox* shows more prominent deformation than *S. fatalis*, while during pull-back and lateral-shaking, the deformation in *S. fatalis* is more pronounced.

When the canines alone are considered, the teeth of *T. atrox* consistently experience lower von Mises, tensile and compressive stresses in all tested scenarios. Quantitative stress magnitudes obtained from equidistant nodes along the canines confirm the pattern seen in the contour plots. It is noteworthy that the canines of *T. atrox* show a distinct region across the whole length of the tooth on the lateral surface, corresponding to the triangular ridge of the tooth that has only very low or no stress magnitudes.

**Figure 7:**
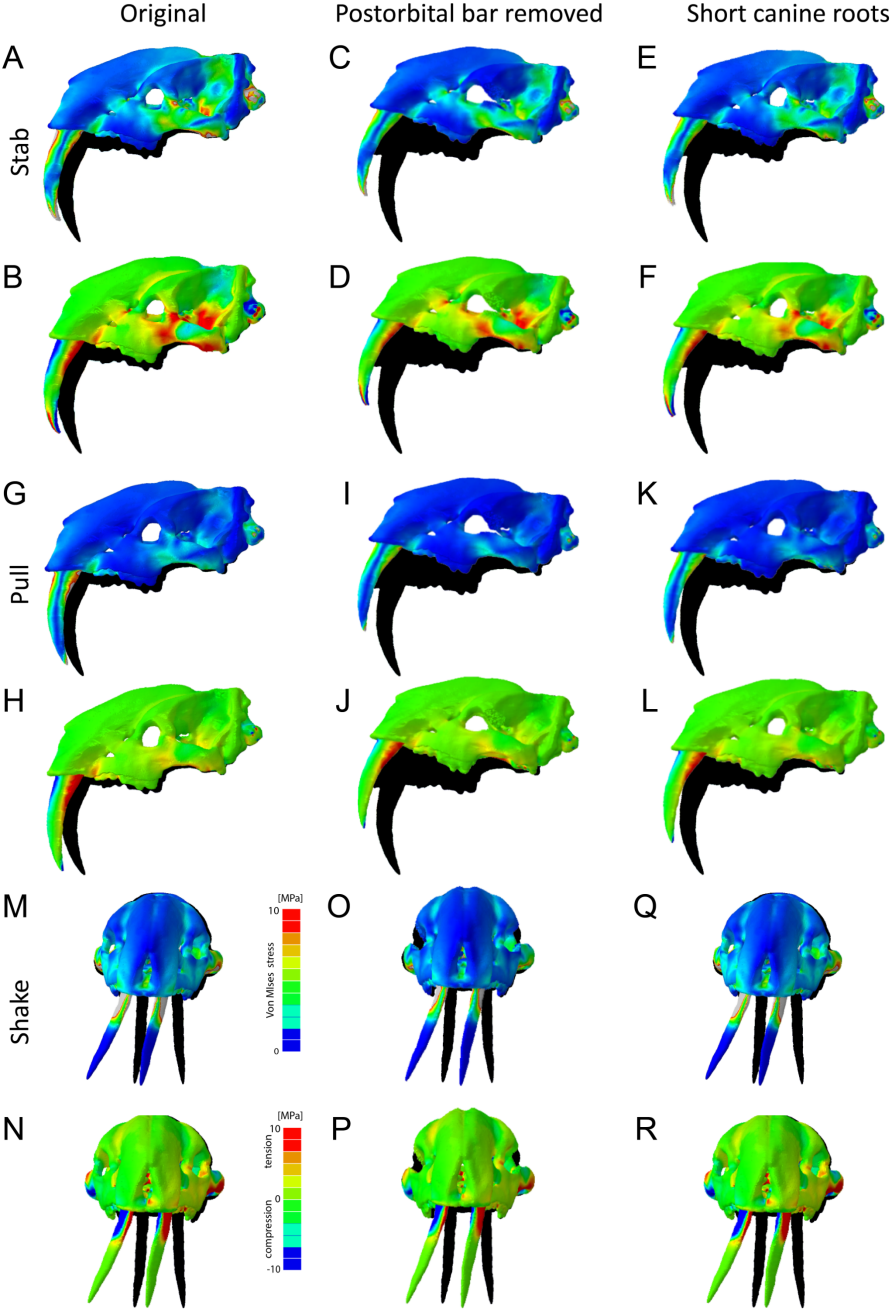
Finite element analysis results for different hypothetical models of *Thylacosmilus atrox*. Model of the original skull (A, B, G, H, M, N), model with postorbital bar removed (C, D, I, J, O, P) and model with shortened canine tooth roots (E, F, K, L, Q, R). For each scenario deformed skull models with von Mises stress contour plots (A, C, E, G, I , K, M, O, Q) and tensile/compressive stress plots (B, D, F, H, J, L, N, P, R) are superimposed on the original, undeformed shape shown in black. Deformation is exaggerated by a factor of 100.

In addition to the original skull morphologies, two hypothetical models of *T. atrox* were analyzed to test the functional contribution of osteological modifications found in *T. atrox* but not in *S. fatalis*: the postorbital bar and the elongated canine tooth roots extending dorsally and posteriorly to the orbits (Fig. 7). For the first hypothetical model, the postorbital bar was digitally removed. Results show that the removal of the postorbital bar increases the deformation of the skull during stabbing and pulling scenarios substantially compared to the original morphology. Von Mises and tensile stresses are increased in the zygomatic arch and slightly in the lateral braincase wall in both scenarios. For the lateral shake scenario, the removal of the postorbital decreases deformation but increases von Mises stress slightly in the maxilla.

For the second hypothetical model, the canine tooth roots were shortened to the length of those in *S. fatalis* and the empty root canal was digitally filled in and assigned the material properties of the surrounding bone. Similarly, as with the removal of the postorbital bar, the shortening of the roots results in an increase in deformation of the skull in the stabbing and pulling scenario. However, the effect on stress magnitudes is negligible. For the lateral shake scenario, the effect of shortening the tooth roots is not different from the original morphology.

### Dental microwear texture analysis

**Figure 8:**
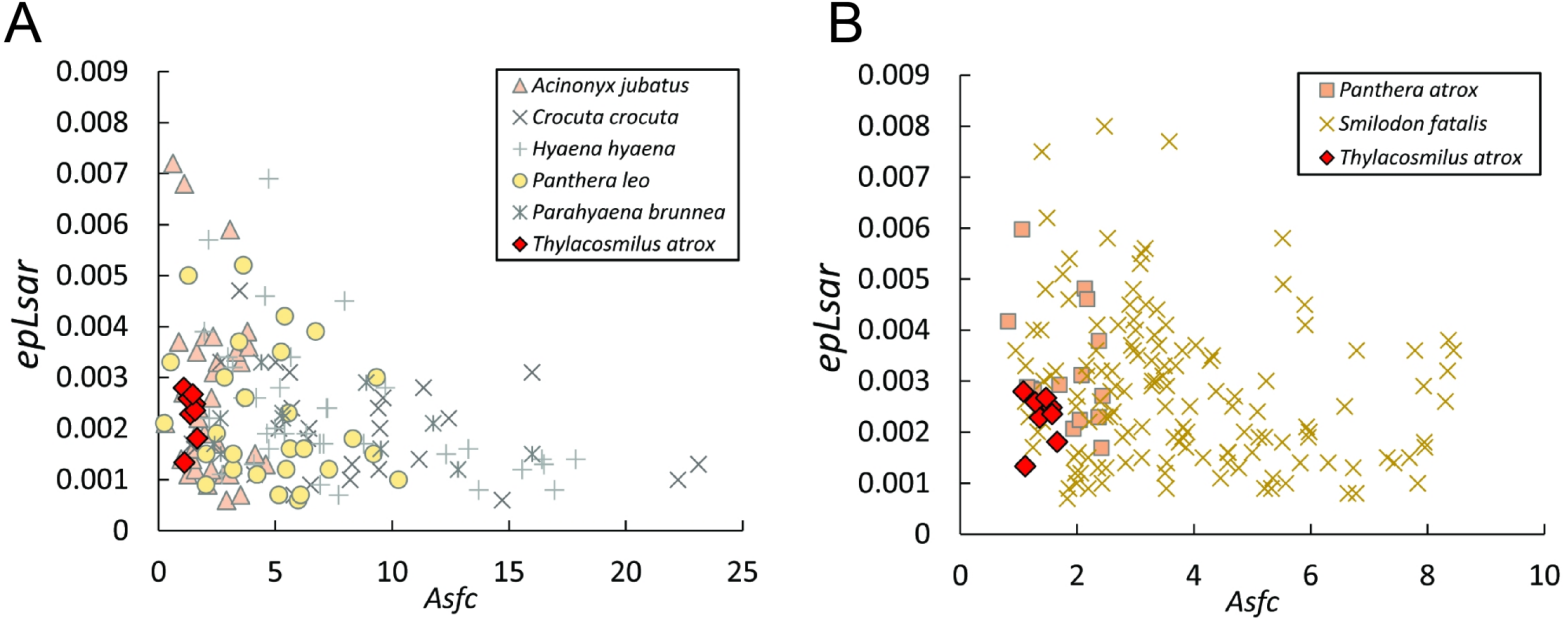
Scatter plots of dental microwear results. Scatter plots of dental microwear attributes complexity (*Asfc*) and anisotropy (*epLsar*) of *Thylacosmilus atrox* (red) in comparison to A, extant and B, extinct taxa. Specimens of *T. atrox* demonstrate low *Asfc* and low *epLsar* values, indicative of neither tough nor hard food consumption, suggestive of a soft-food diet of primarily of fresh flesh and/or soft organs. Data from taxa other than *T. atrox* are from DeSantis (2018), DeSantis et al. (2012), DeSantis & Patterson (2017) and DeSantis et al. (2019).

**Figure 9:**
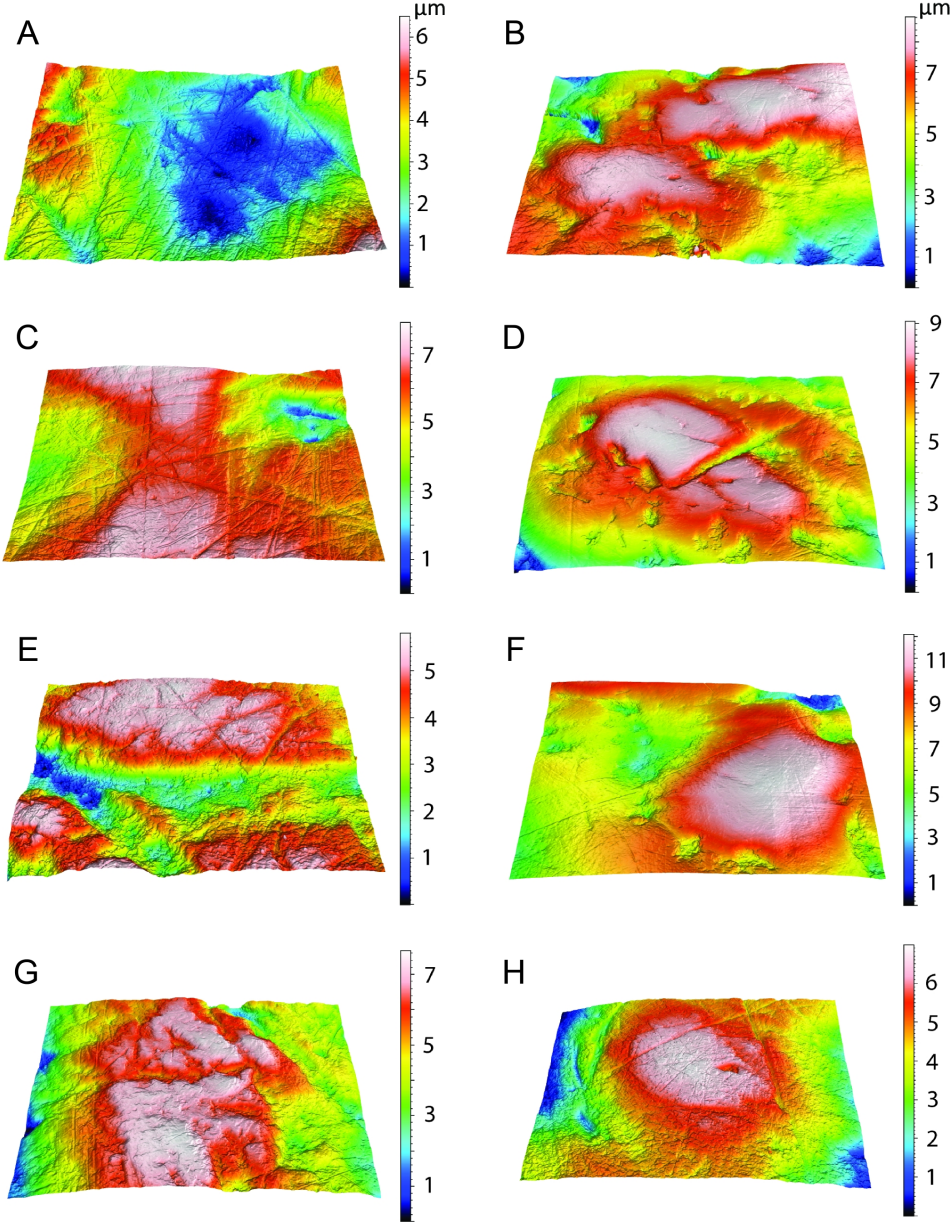
Results of the dental microwear texture analysis. Dental microwear surfaces of all teeth examined of *Thylacosmilus atrox* from FMNH P14344 (lm2, lm3, lm4, A,C,and E, respectively) and FMNH P14531 (LM2, RM1, RM2, RM3, RM4, G, B, D, F, and H, respectively).

**Figure 10:**
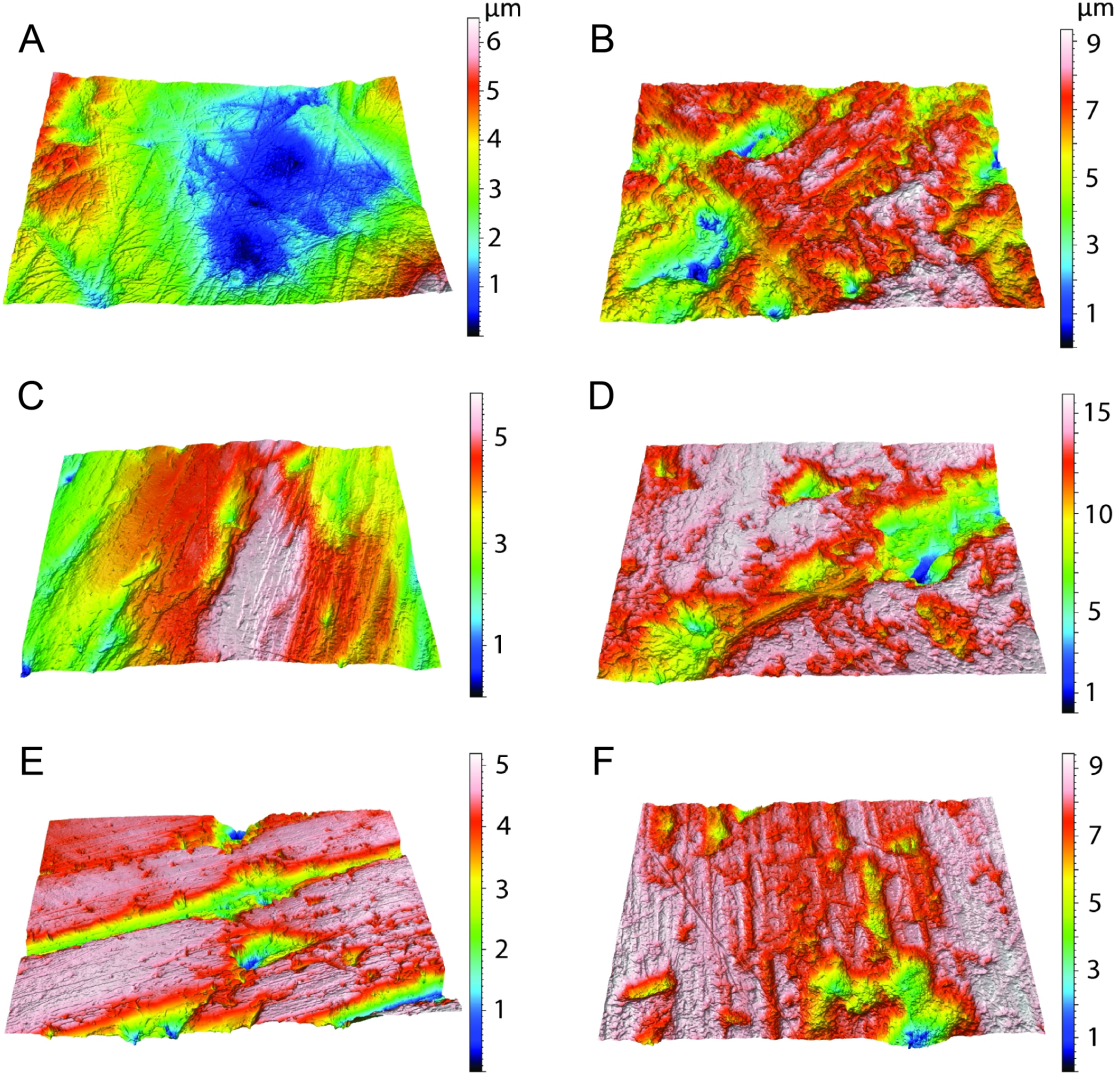
Example microscopic wear surfaces of extant and extinct taxa used for comparison. A. *Thylacosmilus atrox* (FMNH P14344). (B) Crocuta crocuta (NMNH 182085, the spotted hyena). (C)Acinonyx jubatus (AMNH 119657, the cheetah). (D) Hyaena hyaena (FMNH 101982, the striped hyena)., (D)Panthera leo (AMNH 52073, the lion). (F) Smilodon fatalis (LACMHC 2002-L-143, extinct saber-tooth cat).

The data from *Thylacosmilus atrox* are from two individual specimens and span upper and lower molars (see Figs. 8, 9 and 10 and Table 4). There are no systematic differences between teeth more anterior and more posterior in the jaw, suggesting that these “carnassial-like” teeth likely serve similar purposes throughout the jaw (as is also the case in other marsupial carnivores such as the Tasmanian devil, *Sarcophilus harrisi*). Complexity values range from 1.080 to 1.659, with a mean of 1.388 (standard deviation, *n* − 1, SD = 0.216). Anisotropy values range from 0.0013 to 0.0028 with a mean of 0.0023 (SD = 0.0005). Statistical comparisons with other taxa must be interpreted with caution, as all samples of *T. atrox* are from one to two individuals in contrast to the extant and extinct taxa from DeSantis et al. (2012), DeSantis & Patterson (2017), DeSantis et al. (2019) and DeSantis (2018) which largely represent unique individuals.

The complexity values of *T. atrox* (see Figs. 8, 9 and 10) are indistinguishable from those of the cheetah (*Acinonyx jubatus*: *p* = 0.138), which is known to eat primarily flesh, and the extinct American lion (*Panthera atrox*: *p* = 0.515), which has been inferred to be solitary and to consume primarily fresh flesh (DeSantis et al., 2012). In contrast, *T. atrox* has significantly lower complexity values than other extant carnivorans, including the lion (*Panthera leo*: *p* < 0.0001), hyenas (*Crocuta crocuta*, *Hyaena hyaena, Parahyaena brunnea*: for all *p* < 0.0001), and extinct *S. fatalis* (*p* = 0.001) from the La Brea Tar Pits in southern California (DeSantis et al., 2012; DeSantis et al., 2019) and Friesenhahn Cave in Texas (DeSantis, 2018). It should be noted that all *T. atrox Asfc* values are also significantly less than captive *P. leo* values (*p* = 0.002; mean = 3.266 ± 1.469 standard deviation; range of 2.092–5.667; DeSantis & Patterson, 2017), the captive lions which were typically fed soft foods like horsemeat and beef.

Anisotropy is less telling among extant taxa, with no statistical differences between those compared here. Similarly, *T. atrox* is statistically indistinguishable in anisotropy from all other extant and extinct taxa, although Fig. 8 shows that its values are generally lower than those of the cheetah and *P. atrox*. Only *P. atrox* has significantly higher anisotropy than the extant feliforms (all *p* < 0.02), suggesting that it ate tougher food than these taxa. *T. atrox* does exhibit significantly higher *epLsar* values than captive lions (*p* = 0.023), though mean values for both are low (0.0023 and 0.0015, respectively).

## Discussion

### Unique craniodental aspects of *Thylacosmilus atrox* revealed by our analyses

The correspondence analysis shows that *T. atrox* has a unique combination of craniodental traits, making it unlike any known placental sabre-toothed carnivore (Fig. 5). As previously noted, none of these features can be ascribed to it being a metatherian rather than a eutherian.

The biomechanical analysis shows that the craniodental resistance to stresses in predatory scenarios is significantly different between *T. atrox* and *S. fatalis* (Fig. 6, Table 3). In general, *T. atrox* was more resistant to stresses generated by pulling back with the canines and with lateral deflection of the canines, but less resistant to stresses generated by stabbing (Fig. 6). Indeed, the performance of *T. atrox* in these pull back and lateral deflection scenarios appears to be superior to other dirk-toothed machairodonts, and comparable to the scimitar-toothed saber-tooth *Homotherium serum* and the conical-toothed *P. leo* (Figueirido et al., 2018). The canines of *T. atrox* were especially stress-resistant (Fig. 6), rendered so by their unique sub-triangular shape forming a ridge along the lateral surface (Figs. 2A and 3F). In addition, the analysis of the hypothetical models demonstrates that specific osteological modifications in the skull of *T. atrox* increased cranial stability, especially in simulated pull-back action (Fig. 7). In particular, the presence of the postorbital bar braces the skull against deformation.

The dental wear analysis shows that *T. atrox* had a preference for soft food, similar to the cheetah, which consumes only meat, and no bone (Phillips, 1993). However, we note that the lack of incisors in *T. atrox* would make tearing meat off the bone difficult, if not impossible (Biknevicius, Van Valkenburgh & Walker, 1996): thus *T. atrox* may have exhibited unique dietary behavior with no analog to extant taxa. We discuss below how these differences between *T. atrox* and feloid carnivores (extant and extinct) might relate to differences in predatory behavior and food consumption.

### The possible lifestyle of *Thylacosmilus atrox*

While the superficial appearance of *Thylacosmilus atrox* resembles that of placental saber-tooths, its detailed anatomy makes this animal an ecomorphological puzzle, and the analyses performed here show it to be unlike other carnivores, saber-toothed or otherwise. While we can demonstrate that *T. atrox* could not have been a predator in the mode proposed for the saber-toothed feliform carnivorans, it is challenging to propose an alternative mode of life. We note that, while there is often the temptation to shoehorn an extinct animal into the ecomorphological role of an extant one (see Figueirido, Martín-Serra & Janis, 2016)—or even, as in this case, the proposed ecomorphological role another extinct animal—*T. atrox* may well have had no analogs in the extant or extinct fauna. We extend this discussion of extinct animals without living analogs in the conclusions. Here we present some ecomorphological hypotheses for *T. atrox* that align with the peculiarities of its anatomy.

The upper canines of *T. atrox* are superficially similar to those of a dirk-toothed felid; but the greater degree of canine protrusion, the slight divergence at the tip, and the subtriangular profile, imply a different usage from those of placental saber-tooths. We note that the unusual subtriangular shape of these canines makes them appear more like a claw than a blade; and, like a claw, they appear well-adapted for pulling back. Our biomechanical study shows that both the skull and the canines of *T. atrox* are better in resisting pull back stresses than those of *S. fatalis*. Did *T. atrox* preferably use these canines for pulling back activity, i.e., disemboweling carcasses, rather than killing its prey by stabbing? All large carnivores today use their canines for both stabbing and tearing: indeed, both types of canine use would be necessary to feed on a carcass. However, the peculiar mode of strengthening the canines of *T. atrox* may reflect a particular specialty for carcass opening. The very large gape noted for *T. atrox* might also have been useful in such proposed behaviors, allowing its mouth to encompass the bellies of the prey (see Churcher, 1985).

We note in the context of precise canine placement the small infraorbital foramen, which contrasts with the large foramen of placental saber-tooths (and also extant large felids). The infraorbital foramen is for the passage of the infraorbital nerve (cranial nerve V_2_), which transmits somatosensory information from the snout region. A large foramen might reflect the possession of a large nerve, and has been interpreted as indicative of a high degree of sensory feedback from the muzzle (Mivart, 1881), making it an important component in the accurate positioning of the canines during the killing bite, especially important if dealing with struggling prey (Antón, 2013). The small infraorbital foramen of *T. atrox* supports the hypothesis that its canines were not used for killing prey, as it would not require such careful and precise positioning of the canines.

The postcanine teeth of *T. atrox* exhibit blunted tip wear, unlike the shearing wear on the teeth of carnivores that specialize on flesh (Fig. 4). Yet *T. atrox* was clearly not a bone-crusher: this type of diet is contraindicated by the DMTA analysis and the lack of cranial specializations (including evidence for powerful jaw adductors) seen in extant bone-crushers (Figueirido, Tseng & Martín-Serra, 2013). Churcher (1985, p. 215) noted that the blunted wear on the tips of the postcanine teeth in *T. atrox* resembles the wear on the teeth of thylacines (*Thylacinus cynocephalus,* a modern marsupial carnivore, although now considered extinct). Thylacines also had a weak bite and are reported to have specialized on the internal organs of their prey (Attard et al., 2011). Could this have been the preferred diet of the marsupial saber-tooth?

The cranium of *T. atrox* resembles that of placental saber-tooths in modifications for a wide gape and dorsiflexion: but these actions, with the well-developed mental process, would be necessary to deploy the canines for any action, not necessarily for a predatory attack, and *T. atrox* had less powerful jaw adductor and head depressor muscles than placental saber-tooths (Wroe et al., 2013). *T. atrox* evidences powerful neck musculature; but the points of muscle origins and insertions indicate that their primary function was for stabilization of the head on the neck, perhaps also for resisting torsion. The cervical and caudal cranial anatomy are not indicative of the ability for extreme head elevation and forceful head depression, as observed in the anatomy of placental saber-tooths, implicated in those carnivores for a predatory head strike. However, strong stabilization of the head on the neck, and a head held more horizontally in line with the neck (as indicated by the dorsally-positioned occipital condyles), would be advantageous for a predatory scenario for *T. atrox* where the canines were being employed primarily in a pullback mode. The post-orbital bar of *T. atrox*, lacking in all placental saber-tooths with the exception of *Barbourofelis*, may have been important for resisting stresses generated by this action (see Fig. 7).

The virtual absence of incisors (certainly the absence of a stout incisor battery) in *T. atrox* is challenging for the hypothesis of a cat-like mode of feeding, as it would have been unable to strip flesh from a carcass or transport its prey. Extant felids use their incisors for these functions, and the incisors of placental saber-tooths are enlarged and procumbent, permitting these behaviors when hypertrophied canines would have limited access by the anterior dentition (Biknevicius, Van Valkenburgh & Walker, 1996). Emerson & Radinsky (1980, p. 307) noted that the loss of incisors is “—puzzling, for there is no other obvious mechanism for grasping and tearing off pieces of prey”. Churcher (1985) also noted this problem, and proposed that the lower canines acted against some structure in the upper jaw (possibly retained small upper incisors), which could also account for the canine wear; but this hypothesis represents a *post facto* compensation, and does not account for the original cause of incisor reduction or loss. Other authors have concluded that the large canines somehow compensated for incisor loss (e.g., Argot, 2004; Turnbull, 1978); however, canines alone cannot be used to prehend food, and enlarged canines only make this proposed role more unlikely.

The absence of incisors in *T. atrox* remains a paleobiological conundrum, for which no author has proposed a positive advantage. We here advance an admittedly speculative proposition for selective factors that could have led to incisor loss in this animal. Incisor loss or reduction in mammals is correlated with the use of a protrusible tongue in feeding, as seen in myrmecophageous mammals (Davit-Béal, Tucker & Sire, 2009). In the walrus the absence of incisors in combination with a vaulted palate allow for the use of a large tongue in suction feeding (Gordon, 1984). We note that the palate of *Thylacosmilus* is somewhat vaulted (see Fig. 3E), and propose that the vaulted palate and the absence of incisors could together indicate the possession of a large tongue, deployed in feeding to intake guts and other internal organs from the opened carcass (Iwasaki, Erdogan & Asami, 2019), note the importance of the tongue in feeding in extant carnivorous mammals). The tough, ductile but amorphous texture of the internal organs and gut contents of the prey, requiring crushing with the teeth rather than shearing, and resulting in abrasive wear rather than attrition, might explain the observed blunted tip wear on the postcanine teeth of *T. atrox*. Clearly, further examination of the macroscopic dental wear of *T. atrox* would be informative, especially the examination of a greater diversity of specimens, as well as a more detailed comparison with the dental wear of *Thylacinus cynocephalus*, which is reported to have had the diet that we propose for *T. atrox* (Attard et al., 2011).

Churcher (1985) appears to be the only author to have speculated on the reason for the jaw symphysis being ligamentous rather than bony. His proposition that this allows for a degree of independent motion of the lower jaws so that the upper canines can be positioned against a sharpening genial pad deserves further investigation. The postorbital bar may be reminiscent of the condition in *Barbourofelis*, but as noted by Emerson & Radinsky (1980), it is unlikely to have served the same function.

The postcranial anatomy of *T. atrox* provides few clues as to its probable predatory behavior. The skeleton is that of a rather generalized robustly-built carnivore: *T. atrox* had relatively short limbs, short (and spreading) metapodials in particular; a foot stance that was semi-digitigrade in the forelimb and plantigrade in the hind limb; a forelimb longer and more powerful than the hindlimb, retaining the ability for considerable pronation and supination; and a short and stiff lumbar region (Argot, 2004; Churcher, 1985; Ercoli, Prevosti & Álvarez, 2012). The overall morphology is suggestive of a bear-like ambulatory mode of locomotion, rather than a felid-like cursorial one (Ercoli, Prevosti & Álvarez, 2012), and it appears that *T. atrox* lacked the ability either to pursue its prey over distance, or to make rapid lunges for ambush predation. *T. atrox* did possess powerful forelimbs, which might have been useful in stabilizing the front of the body in the proposed pullback feeding scenario, whether planted on the ground or on the carcass of the prey; but, in contrast to placental saber-tooths, it lacked retractile claws, although it had a semi-opposable pollux (Argot, 2004; Ercoli, Prevosti & Álvarez, 2012).

Emerson & Radinsky (1980) proposed that retractile claws would have been essential in placental saber-tooths for the immobilization of prey prior to targeting an accurate strike with canines that would have been susceptible to breakage. Churcher (1985) proposed that *T. atrox* would not have required retractile claws: he interpreted the procumbent orientation of the upper canines as enabling *T. atrox* to stab its prey at almost right angles to the body, to penetrate the barrel of a prey item of similar size to a deer or a sheep. Churcher (1985) posited that *T. atrox* hunted by knocking over the prey and then using its body weight to hold it down, with the orientation of the upper canines obviating the need for retractile claws to stabilize the prey for an accurate kill. Note, however, that the stiff lumbar anatomy would have limited the agility of *T. atrox* in this predatory scenario. The lack of retractile claws in *T. atrox* remains problematical for proposals of the type of predatory behavior proposed for placental saber-tooths.

## Conclusions

Although some earlier researchers interpreted *Thylacosmilus atrox* as a scavenger (see Marshall, 1976; Riggs, 1934), since the mid 20th century this animal has been interpreted as a fearsome predator, perhaps even more specialized for a saber-toothed lifestyle than its placental counterparts (e.g., Wroe et al., 2013). Although the canines of *T. atrox* appear to dwarf those of placental saber-tooths, leading to the interpretation of its lifestyle as one of a super saber-toothed predator, we consider that these magnificent teeth may have over-influenced scenarios of *T. atrox’* s behavior. We propose here *T. atrox* has been “shoehorned” (see Figueirido, Martín-Serra & Janis, 2016) into the saber-tooth ecological role: much less attention has been paid to the way in which this animal differs in its morphology from placental saber-toothed predators, making a similar type of predatory behavior unlikely.

As noted earlier, an extinct animal may not have any ecomorphological analog, extant or extinct. Indeed, if elephants or giraffes were known only from their bones, it would be necessary to propose a mode of life different from any other known animal, perhaps to the incredulity of the audience. Figueirido, Martín-Serra & Janis (2016) noted several cases of extinct mammals proposed to have a different mode of life from any known today, including a unique mode of swimming locomotion for the early cetacean *Ambulocetus* (Thewissen & Fish, 1997) and bipedal striding in sthenurine kangaroos (Janis, Buttrill & Figueirido, 2014, this hypothesis now verified by trackways, see Camens & Worthy, 2019), and they proposed a mode of predation for the ‘marsupial lion’ (*Thylacoleo carnifex*) non-analogous to any extant predator (grasping with the teeth and killing with the forelimbs). While our hypothesis about the possible lifestyle of *T. atrox*, one without analog in any known animal, is necessarily speculative, it is nevertheless grounded in an understanding of its unique anatomy. In addition, while no large placental carnivore today is known to survive purely by scavenging, marsupials have a metabolic rate of around two-thirds that of a similarly-sized placental (McNab, 1986). Thus, *T. atrox* would likely have required considerably less food per day than a similar placental carnivore.

Biomechanical analyses indicate superior ability of *T. atrox* to placental saber-tooths in pulling back with the canines, and support the hypothesis that these canines were employed to open carcasses. The deployment of the canines in a predatory head strike, accompanied by precision tooth placement in stabbing, is contradicted by the following anatomical features: neck musculature indicative of head stabilizing and resistance of torsion rather than strong head deflection; a slight divergence of the canines; and a small infraorbital foramen. The lack of retractile claws is also counter-indicative of a predatory scenario of stabilizing a prey item for a predatory head strike, and the limb anatomy shows that *T. atrox* could not have run down its prey. The dental microwear of *T. atrox* indicates a soft diet of meat and/or internal organs, and the lack of an incisor battery would render any long-canined predator unable to tear meat off the carcass, leading us to the proposal that it specialized on the internal organs of the prey, perhaps employing a large tongue for assistance in their extraction.

The anatomy of *T. atrox* provides few clues as to how it might have actually dispatched its prey. We advance the suggestion that it was not an active predator, but rather relied on the use of existing carcasses, deploying its large canines for carcass opening rather than for killing, a hypothesis supported by our biomechanical analyses that show superior performance in a “pull-back” scenario. It is clear that *T. atrox* was not a bone-cruncher, unlike many extant mammalian scavengers; but note that the ecology of the Argentinian Pliocene would have been unlike any in today’s world, where the predominant predators were large, terrestrial birds, the phorusrachiformes. Did their role as top predators perhaps leave a surplus of carcasses?

Although deducing the predatory mode of this strange metatherian is challenging, we can say with some confidence that it did not evince anatomy well-adapted to perform the type of head strike proposed to be the predatory behavior for placental saber-tooths. Certainly, *Thylcosmilus atrox* was a very different type of carnivorous mammal than the placental saber-tooths: the oft-cited convergence with placentals such as *Smilodon fatalis* deserves a rethink, and the “marsupial saber-tooth” may have had an ecology unlike any other known carnivorous mammal.

## Additional Information and Declarations

### Data Availability

The following information was supplied regarding data availability: The digital models used for FEA are available at: Janis, C. M., Figueirido, B., DeSantis, L., & Lautenschlager, S. (2020). An eye for a tooth: Thylacosmilus was not a marsupial ”saber-tooth predator” [Data set]. Peerj. Zenodo http://doi.org/10.5281/zenodo.3813712. The following specimens were examined in this study: *Smilodon fatalis*, LACMRLP R37376 (Los Angeles County Museum of Natural History Rancho La Brea ); *Thylacosmilus atroxin*, FMNH P14531 (Field Museum of Natural History (Chicago, IL, USA)).
